# A Five-Dimensional Network Meta-Analysis of Chinese Herbal Injections for Treating Acute Tonsillitis Combined With Western Medicine

**DOI:** 10.3389/fphar.2022.888073

**Published:** 2022-06-17

**Authors:** Peiying Huang, Yin Li, Bixuan Huang, Shuai Zhao, Li Chen, Hansu Guan, Yan Chen, Yuchao Feng, Xiaoyan Huang, Yi Deng, Sisi Lei, Qihua Wu, Haobo Zhang, Zhongyi Zeng, Linsheng Zeng, Bojun Chen

**Affiliations:** ^1^ The Second Clinical Medical School of Guangzhou University of Chinese Medicine, Guangzhou, China; ^2^ Guangdong Provincial Key Laboratory of Research on Emergency in Traditional Chinese Medicine, Clinical Research Team of Prevention and Treatment of Cardiac Emergencies with Traditional Chinese Medicine, Guangzhou, China; ^3^ The First Clinical Medical School of Guangzhou University of Chinese Medicine, Guangzhou, China; ^4^ Department of Nursing, Hubei University of Arts and Science, Xiangyang, China; ^5^ Emergency Department of Guangdong Provincial Hospital of Traditional Chinese Medicine, Guangzhou, China; ^6^ Emergency Department of the Third Affiliated Hospital of Guangzhou University of Chinese Medicine, Guangzhou, China; ^7^ Shenzhen Traditional Chinese Medicine Hospital, Shenzhen, China

**Keywords:** acute tonsillitis, Chinese herbal injections, western medicine, efficacy, 5-dimensional network meta-analysis

## Abstract

**Background:** Acute tonsillitis has high morbidity. Chinese herbal injections (CHIs) were reported to be useful in treating acute tonsillitis and might reduce the probability of antibiotic resistance. Nevertheless, the optimal strategy for combining CHIs with western medicine (WM) to treat acute tonsillitis remains unclear.

**Methods:** We retrieved data from the following databases with retrieval time from inception to 11 January 2022: PubMed, Embase, Web of Science, Cochrane Library, China National Knowledge Infrastructure, Wanfang Database, Weipu Journal Database, and Chinese Biomedical Literature Database. Version 2 of the Cochrane risk-of-bias tool (ROB2) was used for evaluating the quality of the included studies. R 4.1.2, STATA 14.0, and Python 3.10.4 were employed for network meta-analysis, with 5-dimensional K-means cluster analysis, meta-regression analyses, sensitivity analyses, and subgroup analyses.

**Results:** A total of 110 randomized controlled trials including 12,152 patients were included. All the studies were rated as “high risk” and “some concerns”. In terms of improving clinical effectiveness rate, Qingkailing injection + WM ranked ahead of other interventions (89.51%). Regarding reducing antipyretic time, Reduning injection + WM had the highest-ranking probability (68.48%). As for shortening sore throat relief time, Shuanghuanglian injection + WM ranked first (76.82%). Concerning shortening red and swollen tonsils relief time, Yanhuning injection + WM possessed the highest-ranking probability (89.17%). In terms of reducing tonsillar exudate relief time, Xuebijing injection + WM ranked ahead of the other interventions (94.82%). Additionally, the results of the cluster analysis suggested that Xuebijing injection + WM, Reduning injection + WM, and Yanhuning injection + WM were probably the best interventions. Furthermore, adverse drug reactions rate of Xuebijing injection + WM, Reduning injection + WM, Yanhuning injection + WM, Qingkailing injection + WM, and Shuanghuanglian injection + WM were individually 0.00%, 3.11%, 3.08%, 4.29%, and 4.62%.

**Conclusions:** CHIs + WM have a better impact on patients with acute tonsillitis than WM alone. Xuebijing injection, Reduning injection, and Yanhuning injection might have potential advantages in treating the disease. Concerning adverse drug reactions, Xuebijing injection is presumably the optimal CHI. More high-quality studies are needed to further confirm our findings.

**Systematic Review Registration:** CRD42022303243; URL= https://www.crd.york.ac.uk/PROSPERO/display_record.php?RecordID=303243

## Introduction

Acute tonsillitis is a type of acute upper respiratory tract infection, with acute sore throat as the principal symptom, accompanied by fever, red and swollen tonsils, enlarged cervical lymph nodes, and may be associated with tonsil exudation (Bartlett et al., 2015; Windfuhr et al., 2016). The disease affects both sexes and all age groups, predominantly in school-aged children (Sidell and Shapiro, 2012). It is estimated that acute tonsillitis makes up approximately 1.3% of outpatient visits (Kocher and Selby, 2014), which generates a substantial workload for primary care physicians and places huge financial pressures on medical budget (Bird et al., 2014).

In 50%–80% of acute tonsillitis patients, the causative pathogens are viruses (e.g., Epstein–Barr virus, rhinovirus, respiratory syncytial virus, adenovirus, and coronavirus), while 5%–36% of cases are caused by bacteria, for the most part, Group A beta-haemolytic streptococci (Ebell et al., 2000; Bird et al., 2014). In western medicine (WM), symptomatic and supportive treatment is the mainstay of viral cases, such as fluid rehydration, antipyretic analgesics, local anesthetics, and corticosteroids. Antibiotics are used as prescribed when there is a possibility of bacterial infection. Although pathogen detection and Centor score/McIsaac score are helpful in the pathogen diagnosis, it remains difficult to distinguish between a bacterial or viral etiology clinically (Bird et al., 2014; Windfuhr et al., 2016). Standard use of antibiotics, therefore, is difficult to achieve for clinicians, which may bring the promotion of bacterial resistance as well as adverse drug reactions. Additionally, antipyretic analgesics, anesthetics, or corticosteroids are classically restrained in some patient populations due to their certain side effects, such as non-steroidal anti-inflammatory drugs in gastrointestinal bleeding and opioids in airway compromise (Bird et al., 2014). Tonsillectomy, a way to deal with recurrent acute tonsillitis, also be restrained during the acute phase and has limited long-term benefits (Morad et al., 2017).

Compared with WM, Traditional Chinese medicine presents the following advantages in treating acute tonsillitis: multiple mechanisms of action (e.g., antimicrobial, anti-inflammatory, antipyretic, pain relief), few contraindications, and low-cost treatment (Fan et al., 2017). Chinese herbal injections (CHIs) are intravenous injections prepared by extracting the active ingredients of traditional Chinese medicine, with the characteristics of rapid onset and improved bioavailability. Research showed that contrasted with WM alone, CHI combined with WM has better clinical efficiency, including more preferable relief of symptoms and shorter disease duration, which may reduce the adverse drug reactions of WM and decrease the risk of antibiotic resistance (Zhou et al., 2020). However, there is a wide variety of CHIs used for acute tonsillitis with few studies comparing them. We thus initiated a network meta-analysis to achieve these comparisons.

## Methods

This study was conducted following the PRISMA extension statement (Hutton et al., 2015) with a PRISMA checklist which is provided in Supplementary File S1.

### Search Strategy

We searched relevant databases including PubMed, Embase, Web of Science, Cochrane Library, China National Knowledge Infrastructure, Wanfang Database, Weipu Journal Database, and Chinese Biomedical Literature Database from database inception to 11 January 2022. The search strategies are provided in Supplementary File S2.

### Study Selection

Only randomized controlled trials (RCTs) which targeted the treatment of CHIs to acute tonsillitis were included. In the selected studies, CHIs plus WM should be compared with WM alone or/and another type of CHIs plus WM. Notably, each group within one included trial received the same treatment regimen of WM. No limitations were defined by age, sex, or race whereas patients with concurrent acute tonsillitis and infections at other sites (e.g., pneumonia) were excluded. The outcome of interest included clinical effectiveness rate (proportion of patients improving after treatment), antipyretic time, sore throat relief time, red and swollen tonsils relief time, tonsillar exudate relief time, and adverse drug reactions (ADRs). A study was admitted according to the inclusion criteria independently by two reviewers. Discrepancies were resolved by consensus between the two reviewers or arbitrated by a third reviewer.

### Data Extraction and Quality Assessment

Data regarding trial information (title, first-author, publication year, sample size, trial duration, interventions, and control), population characteristics (sex, age, and consistency of baseline), reported outcomes (response rate in categorical variables and means/standard deviation in continuous variables), information on methodology (blinding, random methods, and measurement of each indicator), and sponsorship from pharmaceutical companies, were extracted by two independent reviewers using Excel 356 software. The reviewers further used Version 2 of the Cochrane risk-of-bias tool for randomized trials (RoB 2) to assess the risk of bias for each outcome of the included RCTs through the following aspects: randomization process, deviations from intended interventions, missing outcome data, measurement of the outcome, and selection of the reported result (Sterne et al., 2019). Any discrepancies were resolved by discussions between the two reviewers, and if necessary, by arbitration by a third reviewer.

### Data Analysis

In this study, the program was analyzed by a random-effects network meta-analysis within a Bayesian framework (Salanti, 2012; Mavridis and Salanti, 2013). Based on four Monte Carlo Markov Chains, 200,000 in iterations and 10,000 in annealing were set. Risk Ratio (RR)with 95% confidence interval (CI) was calculated as pooled effect measure for categorical variables while pooled effect measures of continuous variables were expressed as Mean Differences (MD) with 95%CI. A league table was generated to present the comparisons between each pair of interventions within each outcome. Surface under the cumulative ranking area curves (SUCRA) with mean ranking probabilities were used to summarize treatment hierarchy (Dias et al., 2013). Additionally, node-splitting method was performed to assess the inconsistency of the model by separating evidence on a particular comparison into direct and indirect evidence in outcome(s) with at least one closed loop (van Valkenhoef et al., 2016). A heatmap was closely employed to measure the contribution degree of each pair of interventions for overall inconsistency. Moreover, Global *I* ^2^-statistic was used to evaluate the heterogeneity of the estimated effect size (Higgins and Thompson, 2002). Network meta-regression in the context of a Bayesian framework was further conducted to examine the potential modification effects for outcome(s) with significant heterogeneity. Furthermore, subgroup network meta-analyses and sensitivity analyses were conducted to assess the robustness of the results and deal with heterogeneity. Comparison-adjusted funnel plots and Egger’s test were used to explore potential publication bias in the outcomes with greater than or equal to 10 RCTs (Begg and Mazumdar, 1994; Stuck et al., 1998).

Additionally, for a comprehensive assessment of treatment effect of CHIs + WM, a 5-dimensional K-means cluster analysis based on the SUCRA values of the selected CHIs + WM within each outcome (clinical effectiveness rate, antipyretic time, sore throat relief time, red and swollen tonsils relief time, and tonsillar exudate relief time) was performed (Wan et al., 1988). Missing values were replaced with the mean of the SUCRA values ​​of each outcome. The steps of clustering were as follows: 1) Randomly selected K objects in the data space as the initial cluster centers. 2) According to the Euclidean distances among the SUCRA values and the pre-set cluster centers, the SUCRA values were divided into the cluster center (category) closest to them. 3) A value of the objective function was calculated using the mean of the SUCRA values in each category. Determine whether the values of the pre-set cluster center and the objective function were consistent. If so, output the result; if not, return to the second step to continue the iteration. Subsequently, principal component analysis (Karhunen-Loeve Transform) was used to convert the results of the 5-dimensional K-means cluster analysis into three dimensions via mapping and then visualize the results on a 3-dimensional axis (Bro and Smilde, 2014).

All analyses were performed using R 4.1.2 (gemtc package: network meta-analysis, heterogeneity, inconsistency, network meta-regression, subgroup analysis, and sensitivity analysis; ggplot2 package: SUCRA graphs), STATA 14.0 (publication bias), and Python 3.10.4 (sklearn package: 5-dimensional K-means cluster analysis, principal component analysis; matplotlib package: visualization of the results of principal component analysis).

## Results

### Study Characteristics

**FIGURE 1:**
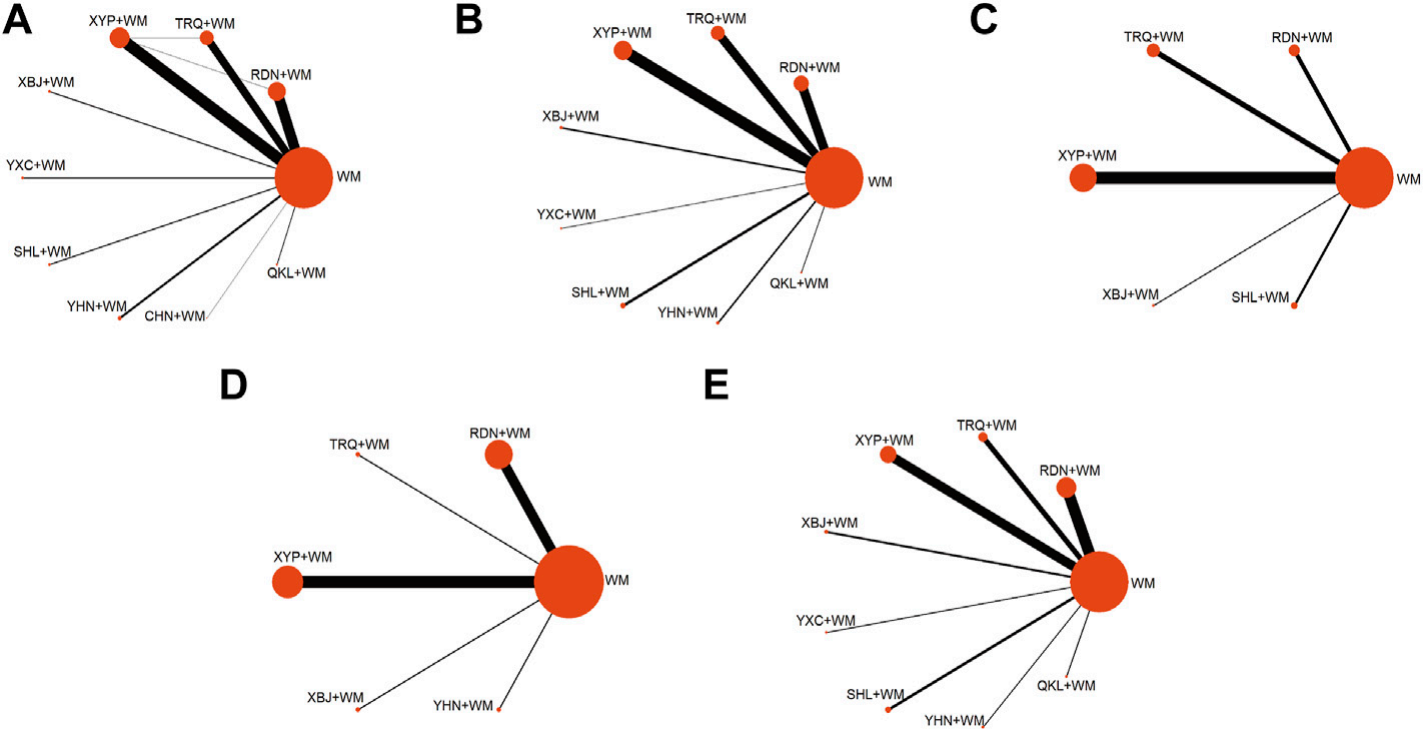
Network graph of different interventions **(A)** Clinical effectiveness rate **(B)** Antipyretic time **(C)** Sore throat relief time **(D)** Red and swollen tonsils relief time **(E)** Tonsillar exudate relief time; WM, Western Medicine; RDN, Reduning injection; TRQ, Tanreqing injection; QKL, Qingkailing injection; XBJ, Xuebijing injection; SHL, Shuanghuanglian injection; YHN, Yanhuning injection; CHN, Chuanhuning injection; YXC, Yuxingcao injection; XYP, Xiyanping injection.

Overall, 869 records were retrieved, in which 110 trials were finally included in the current analysis according to the predesigned criteria (see Supplementary File S3 for the citations of the included studies). A flow chart of the literature search is provided in Supplementary File S4. All the selected trials were two-arm studies with publication years from 1998 to 2021, involving nine kinds of CHIs: Reduning injection (RDN, 31 RCTs), Tanreqing injection (TRQ, 23 RCTs), Xiyanping injection (XYP, 36 RCTs), Yanhuning injection (YHN, seven RCTs), Chuanhuning injection (CHN, one RCTs), Qingkailing injection (QKL, three RCTs), Shuanghuanglian injection (SHL, four RCTs), Xuebijing injection (XBJ, three RCTs), and Yuxingcao injection (YXC, four RCTs) (see Supplementary File S5 for characteristics of the included CHIs). A total of 12,152 patients were included in the entire analysis, of whom 6,772 were male patients (56.78%). Overall, 103 (93.64%), 48 (43.64%), 28 (25.45%), 18 (16.36%), 33 (30.00%), and 44 (40.00%) studies, separately, contributed to the six outcomes, i.e., clinical effectiveness rate, antipyretic time, sore throat relief time, red and swollen tonsils relief time, tonsillar exudate relief time, and ADRs. The selected trails possessed consistent baselines and treatment duration of them ranging from 2 to 10 days. The details of the selected RCTs are shown in Supplementary File S6. Of the 110 RCTs included, the connections among the interventions were visualized as a network diagram within each outcome. The network graphs are depicted in Figure 1, in which the size of node represents the sample size and the thickness of the line between nodes represents the volume of studies.

### Methodological Quality and Risk of Bias Results

**FIGURE 2:**
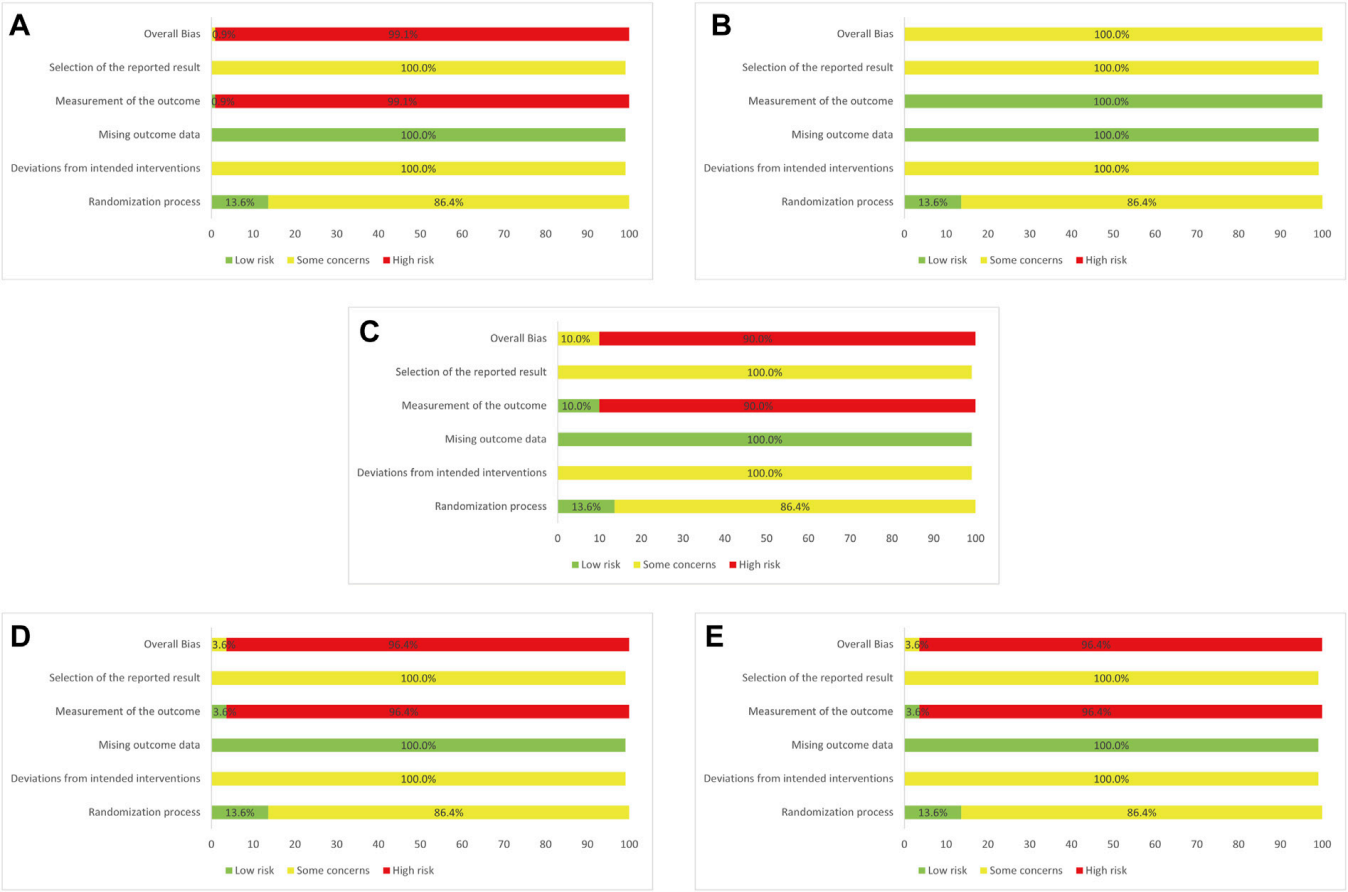
Assessment of risk bias **(A)** Clinical effectiveness rate **(B)** Antipyretic time **(C)** Sore throat relief time **(D)** Red and swollen tonsils relief time **(E)** Tonsillar exudate relief time.

Regarding methodologies of the selected trials, 17 RCTs (15.45%) reported specific details of randomized approaches. Allocation concealment was reported in 13.64% of the cases, and these trials were evaluated as “low risk” in “randomization process”. By contrast, no clear information was reported in all the trials about a predesigned protocol or appropriate analysis that was used to estimate the effect of assignment to intervention, which made both “selection of the reported result” and “deviation from intended interventions” rated as “some concerns”. “Missing outcomes data” was generally a low risk of bias as all the outcomes were comprehensively described with specific number of patients involved in the assessment. Additionally, one RCT (0.91%) showed itself as a double-blind trial while three RCTs (3.60%) blinded trial performers and 10 RCTs (10.00%) blinded trial participants. The differences in blinding among the included trials arrived at the results that in “measurement of the outcome”, severally, 99.1%, 0.00%, 90.00%, 96.4%, and 96.4% of the selected studies in clinical effectiveness rate, antipyretic time, sore throat relief time, red and swollen tonsils relief time, and tonsillar exudate relief time were rated as “high risk” and thus this part of the studies were assessed as “high risk” in “overall bias” (see Figure 2 for risk of bias assessment).

### Network Meta-Analysis

#### Clinical Effectiveness Rate

**FIGURE 3:**
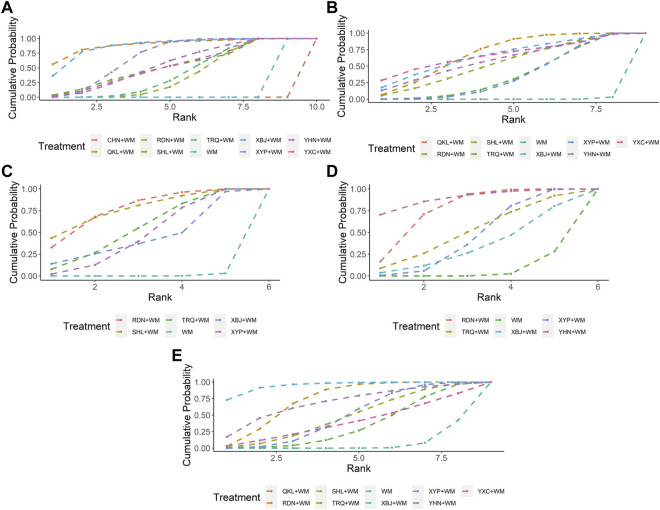
Plots of the surface under the cumulative ranking curves for all interventions **(A)** Clinical effectiveness rate **(B)** Antipyretic time **(C)** Sore throat relief time **(D)** Red and swollen tonsils relief time **(E)** Tonsillar exudate relief time; WM, Western Medicine; RDN, Reduning injection; TRQ, Tanreqing injection; QKL, Qingkailing injection; XBJ, Xuebijing injection; SHL, Shuanghuanglian injection; YHN, Yanhuning injection; CHN, Chuanhuning injection; YXC, Yuxingcao injection; XYP, Xiyanping injection.

Nine CHIs (QKL, RDN, SHL, TRQ, XBJ, XYP, YHN, YXC, and CHN) were involved in the evaluation of clinical effectiveness rate. An improved effect of clinical effectiveness rate was detected for all types of the included CHIs + WM (apart from CHN + WM) vs. WM, while CHN + WM obtained a worse effect than other interventions. XYP + WM improved clinical effectiveness rate as compared with RDN + WM. No such evident effect was observed in any other comparison (see Table 1 for between-intervention differences). According to SUCRA, QKL + WM (89.51%), XBJ + WM (87.39%), and XYP + WM (69.15%) ranked first, second, and third, respectively, whereas RDN + WM (38.25%), WM (11.13%), and CHN + WM (0.03%) separately ranked eighth, ninth, and 10th. More details about SUCRA and its rank probability are individually shown in Figure 3 and Table 2.

**TABLE 1:** Relative effect sizes for each comparison.

Interventions	Clinical Effectiveness Rate, RR (95% CI)	Antipyretic Time, MD (95%CI)	Sore Throat Relief Time, MD (95%CI)	Red and Swollen Tonsils Relief Time, MD (95%CI)	Tonsillar Exudate Relief Time, MD (95%CI)
**CHN + WM vs.**					
QKL + WM	**0.02** (**0.01, 0.62)**	—	—	—	—
RDN + WM	**0.02** (**0.01, 0.71)**	—	—	—	—
SHL + WM	**0.02** (**0.01, 0.70)**	—	—	—	—
TRQ + WM	**0.02** (**0.01, 0.71)**	—	—	—	—
XBJ + WM	**0.02** (**0.01, 0.63)**	—	—	—	—
XYP + WM	**0.02** (**0.01, 0.68)**	—	—	—	—
YHN + WM	**0.02** (**0.01, 0.69)**	—	—	—	—
YXC + WM	**0.02** (**0.01, 0.70)**	—	—	—	—
WM	**0.02** (**0.01, 0.81)**	—	—	—	—
**QKL + WM vs.**					
RDN + WM	**1.16 (0.98, 1.40)**	**−0.09 (−1.63, 1.45)**	—	—	**0.73 (−1.00, 2.46)**
SHL + WM	**1.13 (0.92, 1.41)**	**−0.28 (−1.95, 1.38)**	—	—	**0.22 (−1.70, 2.13)**
TRQ + WM	**1.15 (0.97, 1.39)**	**−0.53 (−2.07, 1.01)**	—	—	**−0.07 (−1.89, 1.75)**
XBJ + WM	**1.03 (0.83, 1.28)**	**−0.07 (−1.87, 1.73)**	—	—	1.66 (−0.36, 3.69)
XYP + WM	**1.10 (0.94, 1.33)**	**−0.54 (−2.06, 0.98)**	—	—	**0.24 (−1.50, 1.98)**
YHN + WM	**1.13 (0.94, 1.37)**	**−0.19 (−2.02, 1.64)**	—	—	**0.80 (−1.54, 3.14)**
YXC + WM	**1.13 (0.93, 1.39)**	**0.01 (−2.08, 2.08)**	—	—	**0.01 (−2.33, 2.34)**
WM	**1.32** (**1.12, 1.59)**	**-1.67** (**-3.15, -0.19)**	—	—	**−0.70 (−2.36, 0.96)**
**RDN + WM vs.**					
SHL + WM	0.98 (0.86, 1.10)	−0.19 (−1.08, 0.69)	0.04 (−0.82, 0.89)	—	−0.51 (−1.58, 0.56)
TRQ + WM	0.99 (0.95, 1.04)	−0.44 (−1.07, 0.18)	−0.22 (−0.88, 0.42)	−0.45 (−1.83, 0.94)	−0.79 (−1.69, 0.10)
XBJ + WM	0.89 (0.77, 1.00)	0.02 (−1.10, 1.14)	−0.40 (−1.56, 0.74)	−0.79 (−2.18, 0.61)	0.94 (−0.32, 2.21)
XYP + WM	**0.96** (**0.92, 0.99)**	−0.44 (−1.02, 0.12)	−0.29 (−0.85, 0.27)	−0.53 (−1.20, 0.17)	−0.49 (−1.22, 0.25)
YHN + WM	0.97 (0.90, 1.05)	−0.10 (−1.26, 1.06)	—	0.51 (−0.96, 1.99)	0.07 (−1.65, 1.79)
YXC + WM	0.98 (0.88, 1.08)	0.09 (−1.44, 1.62)	—	—	−0.73 (−2.45, 1.00)
WM	**1.14** (**1.11, 1.18)**	−**1.57** (−**2.01,** −**1.15)**	**−1.40** (**−1.88, −0.93)**	**−1.28** (**−1.79, −0.79)**	**−1.43** (**−1.92, −0.93)**
**SHL + WM vs.**					
TRQ + WM	1.02 (0.90, 1.16)	−0.25 (−1.14, 0.65)	−0.26 (−1.10, 0.58)	—	−0.28 (−1.49, 0.93)
XBJ + WM	0.91 (0.76, 1.08)	0.21 (−1.08, 1.51)	−0.44 (−1.71, 0.83)	—	1.45 (−0.04, 2.96)
XYP + WM	0.98 (0.87, 1.11)	−0.25 (−1.11, 0.61)	−0.33 (−1.09, 0.45)	—	0.02 (−1.07, 1.12)
YHN + WM	0.99 (0.87, 1.15)	0.10 (−1.22, 1.42)	—	—	0.58 (−1.32, 2.49)
YXC + WM	1.01 (0.86, 1.17)	0.29 (−1.37, 1.95)	—	—	−0.22 (−2.12, 1.69)
WM	**1.17** (**1.04, 1.32)**	**−1.38** (**−2.15, −0.61)**	**−1.44** (**−2.15, −0.73)**	—	−0.92 (−1.86, 0.04)
**TRQ + WM vs.**					
XBJ + WM	0.89 (0.77, 1.01)	0.46 (−0.67, 1.59)	−0.18 (−1.32, 0.96)	−0.34 (−2.17, 1.50)	**1.73** (**0.36, 3.12)**
XYP + WM	0.96 (0.92, 1.00)	0.01 (−0.59, 0.58)	−0.07 (−0.59, 0.47)	−0.09 (−1.44, 1.31)	0.30 (−0.62, 1.23)
YHN + WM	0.98 (0.90, 1.06)	0.34 (−0.82, 1.50)	—	0.96 (−0.93, 2.86)	0.86 (−0.95, 2.68)
YXC + WM	0.98 (0.88, 1.09)	0.53 (−1.01, 2.07)	—	—	0.06 (−1.74, 1.88)
WM	**1.15** (**1.11, 1.19)**	**−1.13** (**−1.59, −0.69)**	**−1.18** (**−1.62, −0.73)**	−0.84 (−2.13, 0.45)	−0.64 (−1.38, 0.11)
**XBJ + WM vs.**					
XYP + WM	1.07 (0.95, 1.24)	**−1.13** (**−1.5, −0.76)**	0.11 (−0.97, 1.21)	0.25 (−1.12, 1.66)	**−1.43** (**−2.72, −0.15)**
YHN + WM	1.09 (0.95, 1.28)	**−1.13** (**−1.5, −0.76)**	—	1.30 (−0.61, 3.20)	−0.87 (−2.88, 1.15)
YXC + WM	1.10 (0.94, 1.30)	**−1.13** (**−1.5, −0.76)**	—	—	−1.66 (−3.69, 0.35)
WM	**1.28** (**1.14, 1.48)**	**−1.6** (**−2.64, −0.56)**	−1.00 (−2.05, 0.05)	−0.50 (−1.81, 0.80)	**−2.36** (**−3.53, −1.21)**
**XYP + WM vs.**					
YHN + WM	1.02 (0.94, 1.09)	0.35 (−0.79, 1.49)	—	1.05 (−0.43, 2.49)	0.56 (−1.18, 2.29)
YXC + WM	1.03 (0.92, 1.13)	0.54 (−0.98, 2.05)	—	—	−0.24 (−1.98, 1.50)
WM	**1.20** (**1.16, 1.23)**	**−1.13** (**−1.50, −0.76)**	**−1.11** (**−1.41, −0.82)**	**−0.75** (**−1.24, −0.30)**	**−0.94** (**−1.48, −0.40)**
**YHN + WM vs.**					
YXC + WM	1.01 (0.89, 1.13)	0.19 (−1.63, 2.01)	—	—	−0.80 (−3.13, 1.53)
WM	**1.17** (**1.10, 1.26)**	**−1.48** (**−2.55, −0.41)**	—	**−1.80** (**−3.19, −0.41)**	−1.50 (−3.15, 0.15)
**YXC + WM vs.**					
WM	**1.17** (**1.06, 1.29)**	**−1.67** (**−3.14, −0.20)**	—	—	−0.70 (−2.35, 0.95)

Note: Bold RR/MD (95% CI) indicates a statistically significant difference; RR, risk ratio; MD, mean differences; 95% CI, 95% Confidence Interval; WM, western medicine; RDN, reduning injection; TRQ, tanreqing injection; QKL, qingkailing injection; XBJ, xuebijing injection; SHL, shuanghuanglian injection; YHN, yanhuning injection; CHN, chuanhuning injection; YXC, yuxingcao injection; XYP, xiyanping injection.

**TABLE 2:** Ranking probabilities of surface under the cumulative ranking area curves (SUCRA) for five outcomes.

Interventions	Clinical Effectiveness Rate (%)	Antipyretic Time (%)	Sore Throat Relief Time (%)	Red and Swollen Tonsils Relief Time (%)	Tonsillar Exudate Relief Time (%)
**QKL + WM**	89.51	66.35			39.07
**RDN + WM**	38.25	68.48	76.77	76.14	73.02
**SHL + WM**	53.37	53.81	76.82		47.25
**TRQ + WM**	42.15	35.60	54.58	50.19	33.54
**XBJ + WM**	87.39	65.45	44.62	33.74	94.82
**XYP + WM**	69.15	34.68	46.61	44.63	48.04
**YHN + WM**	56.08	58.84		89.17	68.92
**YXC + WM**	52.95	66.37			39.03
**CHN + WM**	0.03				
**WM**	11.13	0.42	0.61	6.12	6.31

Note: WM, western medicine; RDN, reduning injection; TRQ, tanreqing injection; QKL, qingkailing injection; XBJ, xuebijing injection; SHL, shuanghuanglian injection; YHN, yanhuning injection; CHN, chuanhuning injection; YXC, yuxingcao injection; XYP, xiyanping injection.

#### Antipyretic Time

Eight CHIs (QKL, RDN, SHL, TRQ, XBJ, XYP, YHN, and YXC) were involved in the evaluation of antipyretic time. In this clinical indicator, shortening was statistically significant for QKL + WM, RDN + WM, SHL + WM, TRQ + WM, XBJ + WM, XYP + WM, YHN + WM, and YXC + WM, as compared with WM. In addition, XBJ + WM was superior to XYP + WM, YHN + WM, and YXC + WM. No significant association was found with other comparators (see Table 1 for between-intervention differences). Based on SUCRA, RDN + WM (68.48%), YXC + WM (66.37%), and QKL + WM (66.35%) ranked first, second, and third, respectively, whereas TRQ + WM (35.60%), XYP + WM (34.68%), and WM (0.42%) separately ranked seventh, eighth, and ninth. More details about SUCRA and its rank probability are individually shown in Figure 3 and Table 2.

#### Sore Throat Relief Time

Five CHIs (RDN, SHL, TRQ, XBJ, and XYP) were involved in the evaluation of sore throat relief time. RDN + WM, SHL + WM, TRQ + WM, and XYP + WM statistically reduced sore throat relief time as compared with WM. No such evident effect was observed with other pairwise interventions (see Table 1 for between-intervention differences). According to SUCRA, SHL + WM (76.82%), RDN + WM (76.77%), and TRQ + WM (54.58%) ranked first, second, and third, respectively, whereas XYP + WM (46.61%), XBJ + WM (44.62%), and WM (0.61%) severally ranked fourth, fifth, and sixth. More details about SUCRA and its rank probability are individually shown in Figure 3 and Table 2.

#### Red and Swollen Tonsils Relief Time

Five CHIs (RDN, TRQ, XBJ, YHN, and XYP) were involved in the evaluation of red and swollen tonsils relief time. RDN + WM, XYP + WM, and YHN + WM showed a significant decrease in red and swollen tonsils relief time as compared with WM, whereas no significant association was found in any other comparison (see Table 1 for between-intervention differences). According to SUCRA, YHN + WM (89.17%), RDN + WM (76.14%), and TRQ + WM (50.19%) ranked first, second, and third, respectively, whereas XYP + WM (44.63%), XBJ + WM (33.74%), and WM (6.12%) individually ranked fourth, fifth, and sixth. More details about SUCRA and its rank probability are individually shown in Figure 3 and Table 2.

#### Tonsillar Exudate Relief Time

Eight CHIs (QKL, RDN, SHL, TRQ, XBJ, XYP, YHN, and YXC) were involved in the appraisal of tonsillar exudate relief time. RDN + WM, XBJ + WM, and XYP + WM were associated with a significant reduction in tonsillar exudate relief time as compared with WM. In comparison with XBJ + WM, both TRQ + WM and XYP + WM obtained a worse effect (see Table 1 for between-intervention differences). According to SUCRA, XBJ + WM (94.82%), RDN + WM (73.02%), and YHN + WM (68.92%) ranked first, second, and third, respectively, whereas YXC + WM (39.03%), TRQ + WM (33.54%), and WM (6.31%) separately ranked seventh, eighth, and ninth. More details about SUCRA and its rank probability are individually shown in Figure 3 and Table 2.

#### Adverse Drug Reactions

ADRs were monitored in 44 RCTs (40.00%), of which 24 studies (21.82%) reported the number of affected patients in detail whereas 20 studies (18.18%) presented no ADRs during the treatment. No ADRs were observed in the reported 52 patients using XBJ (0.00%). The ADRs rate for RDN, TRQ, XYP, YHN, YXC, QKL, and SHL were 3.11%, 0.88%, 2.99%, 3.08%, 2.78%, 4.29%, and 4.62%, respectively, without fatal reactions. The ADRs are further detailed in Table 3.

**TABLE 3:** Details of adverse drug reactions.

	Reduning Injection	Tanreqing Injection	Xiyanping Injection	Yanhuning Injection	Yuxingcao Injection	Qingkailing Injection	Shuanghuanglian Injection
Nausea and vomiting	1.29% (10/773)		1.03% (11/1067)		2.78% (2/72)	1.43% (1/70)	3.08% (2/65)
Diarrhea	0.91% (7/773)	0.44% (1/277)	0.47% (5/1067)				
Dizziness	0.26% (2/773)		0.37% (4/1067)				
Rash	0.65% (5/773)	0.44% (1/277)	1.03% (11/1067)	3.08% (2/65)		2.86% (2/70)	1.54% (1/65)
Chills			0.09% (1/1067)				
Total	3.11% (24/773)	0.88% (2/277)	2.99% (32/1067)	3.08% (2/65)	2.78% (2/72)	4.29% (3/70)	4.62% (3/65)

#### 5-Dimensional K-Means Cluster Analysis

**FIGURE 4:**
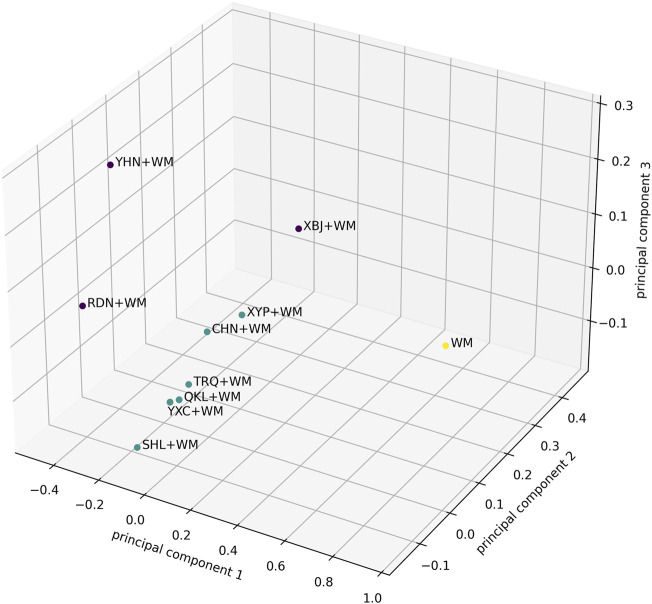
The results of the 5-Dimensional K-means cluster analysis were reduced to three dimensions by principal component analysis. The purple dots represent the best category of curative effect, while the green dots are the second and the yellow dots are the worst. The axes represent the three principal components in the principal component analysis.

A 5-dimensional K-means cluster analysis was conducted to comprehensively compare the effects of the interventions on the five outcomes (clinical effectiveness rate, antipyretic time, sore throat relief time, red and swollen tonsils relief time, and tonsillar exudate relief time). The results reduced to three dimensions by principal component analysis are shown in Figure 4. Upon visual inspection, all interventions were clustered into three categories, in which XBJ + WM, RDN + WM, and YHN + WM were classified as a category with optimal treatment effect while WM alone was as a category with worst curative effect.

#### Inconsistency, Heterogeneity, and Publication Bias

**FIGURE 5:**
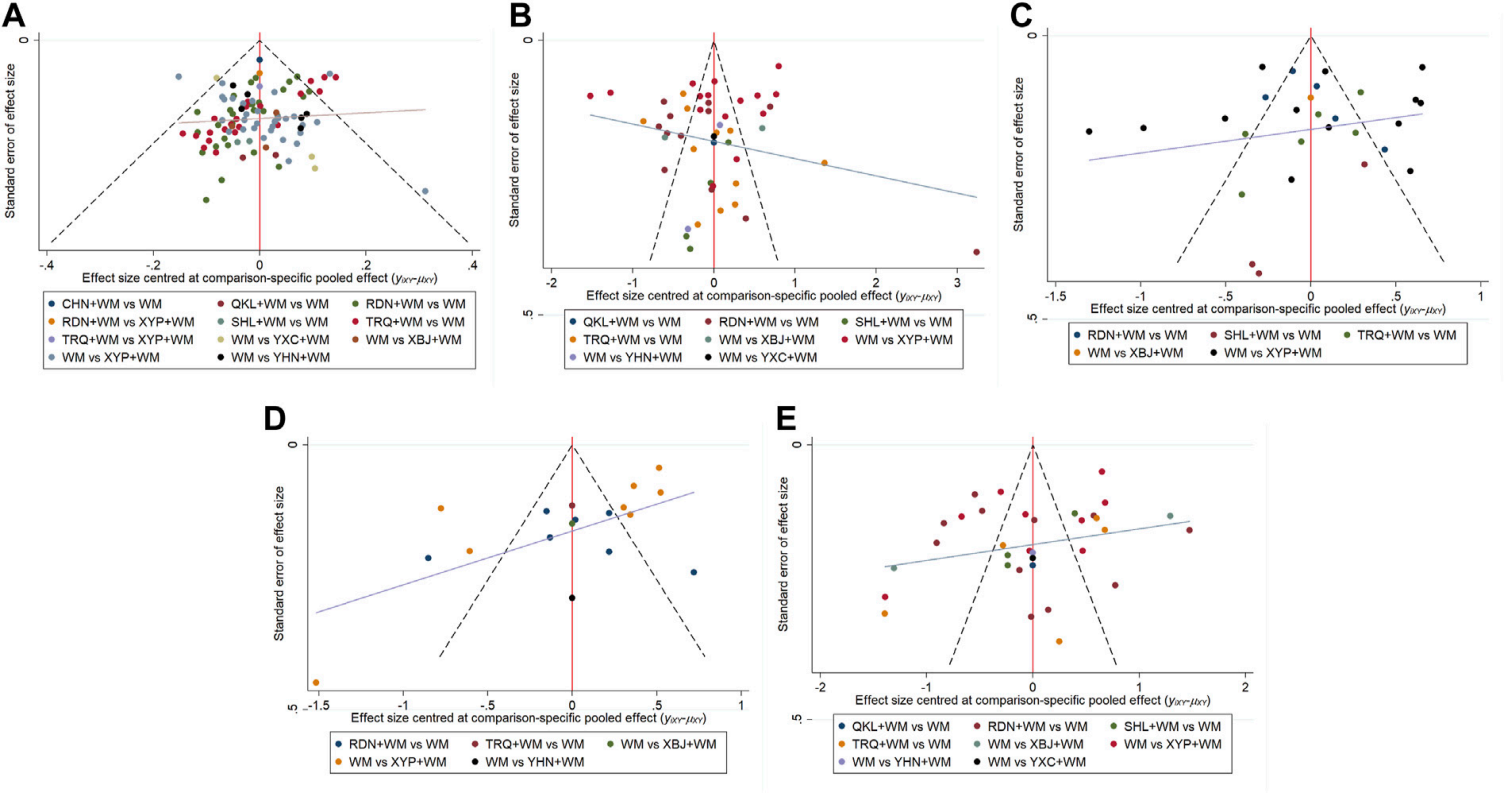
Funnel plots **(A)** Clinical effectiveness rate **(B)** Antipyretic time **(C)** Sore throat relief time **(D)** Red and swollen tonsils relief time **(E)** Tonsillar exudate relief time.

Assessment of inconsistency by node-splitting method indicated that inconsistency was not detected in clinical effectiveness rate as *p*-values in all the comparisons were greater than 0.05 (Supplementary File S7). The heatmap revealed that the pooled effect size in “RDN + WM vs. XYP + WM” had the greatest contribution to the inconsistency of clinical effectiveness rate (see Supplementary File S8 for contribution degree of inconsistency). Regarding heterogeneity, Global *I* ^2^-statistic was 18.07%, 96.54%, 94.12%, 95.96%, and 96.63% for clinical effectiveness rate, antipyretic time, sore throat relief time, red and swollen tonsils relief time, and tonsillar exudate relief time, individually. Upon visual inspection, the funnel plots showed unremarkable asymmetry on both sides of the centerline, which did not suggest a significant risk of publication bias in our sample of the included studies. Nevertheless, quantitative detection of publication bias (Egger’s test) demonstrated that the *p* value for antipyretic time was 0.006 (<0.05), while the *p* values for clinical effectiveness rate, sore throat relief time, red and swollen tonsils relief time, and tonsillar exudate relief time were respectively 0.434, 0.360, 0.424 and 0.400, suggesting that there were small-study effects in the outcome of antipyretic time. The funnel plots are shown in Figure 5 and the results of Egger’s test are provided in Supplementary File S9.

#### Meta-Regression Analyses, Sensitivity Analyses, and Subgroup Analyses

Since there are statistical heterogeneities according to the Global *I* ^2^, four outcomes (antipyretic time, sore throat relief time, red and swollen tonsils relief time, and tonsillar exudate relief time) were analyzed by network meta-regression with patients’ age and publication year as the covariates. The results suggested that red and swollen tonsils relief time would decrease by 0.7 days per year’s change in patients’ age, whereas the covariates were not statistically significant in the remaining outcomes (Supplementary File S10). Sensitivity analysis, a network meta-analysis in selected studies published after 2010, indicated that the overall results were robust (Supplementary File S11). Subgroup analyses were performed according to patients’ age, tonsil suppuration, and treatment regimen of WM. As the number of studies targeting adult patients, patients without suppurative tonsillitis, and patients treated with WM apart from penicillins/cephalosporins was too small to achieve subgroup analyses, the subgroup analyses were finally conducted in studies with pediatric patients, patients with suppurative tonsillitis, patients treated by penicillins, and patients received cephalosporins as the treatment regimen of WM. In the subgroup of children, compared to the overall results, XBJ + WM ranked first in clinical effectiveness rate (78.30%) while QKL + WM was not included in this outcome analysis (Supplementary File S12). The subgroup of patients with suppurative tonsillitis demonstrated that, as compared with the overall results, XBJ + WM ranked first (94.87%) in decreasing antipyretic time (Supplementary File S13). Compared to the overall results, the subgroup for patients who received penicillins as the treatment regimen of WM indicated that XYP + WM ranked first (81.19%) in reducing red and swollen tonsils relief time and YHN + WM possessed the highest-ranking probability (89.78%) in decreasing tonsillar exudate relief time, while XBJ + WM was not included in the tonsillar exudate relief time analysis (Supplementary File S14). In the subgroup for patients who received cephalosporins as the treatment regimen of WM, as compared with the overall results, XBJ + WM ranked ahead of other interventions in the outcomes of clinical effectiveness rate (99.35%) and antipyretic time (95.41%); TRQ + WM ranked first (77.97%) in reducing sore throat relief time; RDN + WM has the highest-ranking probability (85.13%) in decreasing red and swollen tonsils relief time; SHL + WM and YHN + WM were individually not included in the sore throat relief time analysis and red and swollen tonsils relief time analysis (Supplementary File S15).

## Discussion

In the theory of Traditional Chinese medicine, acute tonsillitis is classified as acute nippled moth, which is predominantly caused by pathogenic qi that is associated with heat-toxicity (Gao et al., 2017). Therefore, in the position of the Chinese medicine theory, the main strategy of treating acute tonsillitis is to clear heat and detoxify (Gao et al., 2017). In the current study, all the included CHIs have the efficacy of clearing heat or detoxifying and thus are used for the treatment of acute tonsillitis clinically. Among the CHIs, this network meta-analysis (SUCRA) suggested that QKL, XBJ, RDN, SHL, and YHN might have potential advantages in treating the disease, in which XBJ, RDN, and YHN deserved more attention based on the cluster analysis. Simultaneously, XBJ may be the optimal CHI for acute tonsillitis considering ADRs.

According to our findings, QKL + WM showed good performance in improving clinical effectiveness rate as well as resolving fever through pairwise comparison and ranking probability. QKL is mainly prepared from baicalin, *Isatis tinctoria* L [Brassicaceae], *Lonicera japonica* Thunb [Caprifoliaceae], and *Gardenia jasminoides* J.Ellis [Rubiaceae]. Similar to our study, some studies found that QKL has a significant antipyretic effect, which was associated with repairing the perturbed pathways of lipid metabolism and amino acid metabolism (Gao et al., 2013; Qin et al., 2016). An *in vitro* experiment confirmed an inhibitory efficacy on resistant bacteria containing blaNDM-1 by QKL and especially its active ingredient, baicalin; an experiment also indicated the significant inhibition of QKL plus antibiotics to multidrug-resistant bacteria (Shang et al., 2013). Besides, QKL was confirmed to possess its potent reduction in inhibiting damage caused by infection, e.g., leucopenia and thrombocytopenia (Yi et al., 2021). These pharmacological mechanisms may be tied to the efficacies of QKL to acute tonsillitis.

Apart from QKL, the injections that were prepared from traditional heat-clearing and detoxifying Chinese herbs in our study also included RDN, SHL, and YHN. We found that RDN + WM, SHL + WM, and YHN + WM exerted superior effects in lowering body temperature, shortening sore throat relief time, and reducing red and swollen tonsils relief time, separately. The antipyretic mechanism of RDN might be related to the regulation of biosynthesis as well as sphingolipid metabolism of valine, leucine, and isoleucine (Gao et al., 2020). In addition, RDN was reported to possess anti-inflammatory and antiviral effects (Cao et al., 2015; Xie et al., 2020; Xu et al., 2021), which might work in treating acute tonsillitis. SHL is made from active ingredients of *Forsythia suspensa* (Thunb.) Vahl [Oleaceae], *Lonicera japonica* Thunb [Caprifoliaceae], and *Scutellaria baicalensis* Georgi [Lamiaceae]. Under some *in vitro* and *in vivo* experiments, the injection also had benefit of inhibiting viruses, in which the pathogens might be the perpetrator of acute tonsillitis, e.g., SARS-CoV-2 and influenza A virus H5N1(Tang et al., 2018; Su et al., 2020). Moreover, SHL could inhibit NF-kappaB-mediated production of pro-inflammatory cytokines and chemokines, thereby reducing the inflammatory response to microbial infection (Chen et al., 2002). YHN originates from *Andrographis paniculata* (Burm.f.) Nees [Acanthaceae], a Chinese herbal medicine possessing primary effects of clearing heat and detumescence. Pharmacological research showed that YHN has strong inhibitory effects on respiratory syncytial virus, Coxsackie virus, Epstein-Barr virus, and rotavirus (Liu et al., 2007; Han, 2012; Guan and Cao, 2013; Huang et al., 2013), among which some viruses might cause acute tonsillitis. Additionally, YHN has therapeutic effects on SD rats with upper respiratory tract infection modeled by beta-hemolytic *streptococcus* via inhibiting the expression of IL-1β, IL-6β, and TNF-α(Liang et al., 2012), while beta-hemolytic *streptococcus* is the main bacterium causing acute tonsillitis.

Unlike the CHIs mentioned above consisting of heat-clearing and detoxifying Chinese herbs as raw materials, XBJ, another included intravenous Chinese medicine preparation, derived from a traditional formulation called “*Xuefuzhuyu* Decoction” which does not contain any heat-clearing and detoxifying Chinese herb but Chinese herb activating blood circulation and removing stasis, whereas has the functions of dispelling blood stasis and detoxification. Pharmacological analysis research had demonstrated that the main constituents of XBJ including paeoniflorin, senkyunolide I, safflor yellow A, danshensu, uridine, rosmarinic acid, beta-ocimene-X, gallic acid, protocatechualdehyde, hydroxysafflor yellow A, and oxypaeoniflorin, etc. via ultra-high-performance liquid chromatography (Ji et al., 2010; Jiang et al., 2013), in which the active ingredients play anti-infection and immunomodulatory effects by acting on targets/pathways such as COX-2, IKK-2, 5-LOX, NF-κB, MAPK, eNOS, iNOS, A2AR, and MIF(Ma et al., 2009; Jiang et al., 2013). These pharmacological mechanisms may be related to the treatment of acute tonsillitis with XBJ. Indeed, XBJ has played a vital role in treating sepsis or septic shock as a result of its anti-inflammatory effect as well as immunomodulatory function, and thus the injection has been included in the treatment guidelines of sepsis in China (Branch, 2015). In clinical, XBJ has also been used to treat acute tonsillitis, a disease that is classified as an infectious disease as sepsis. As indicated in our study, XBJ was revealed as the potential optimal CHIs in shortening tonsillar exudate relief time and possibly even the best CHI for the comprehensive treatment of acute tonsillitis, which was consistent with the results of a previous network meta-analysis targeting CHIs plus WM in the treatment of acute tonsillitis in children (involving 65 RCTs as well as six CHIs). In that study, XBJ possessed the highest-ranking probability regarding antipyretic time, sore throat relief time, and red and swollen tonsils relief time, whereas had a similar ranking for clinical effectiveness rate and tonsillar exudate relief time with our subgroup analyses of children (Zhou et al., 2020).

In addition to clinical efficacy, the adverse reactions of CHIs are also attention-worthy. In the current study, we reported both the incidence and types of ADRs for seven CHIs. Although the studies we included did not monitor the occurrence of fatal ADRs, the safety of CHIs remains a concern; how to reduce the occurrence of ADRs in CHIs deserves our attention. Risk factors for ADRs in CHIs, in this case, may provide some recommendations. A retrospective study showed that ADRs are more likely to occur in children or combine with cephalosporin when using QKL (Wu et al., 2018). In addition, ADRs to XBJ are related to vehicle type, dosage, older age, and drug combination (e.g., reduced glutathione, aspirin-dl-lysine, and torsemide) (Wang et al., 2019), while the history of drug allergy, abnormal liver and kidney function, traditional Chinese medicine dialectical medication, dispensing time, drip rate, and drug combination might play roles in ADRs of SHL through multi-factor analysis (Pang and Zhang, 2018). Besides, children are a high-risk group for ADRs with YHN, and off-label drug use is responsible for ADRs in RDN (Huang, 2018; Yu et al., 2019). Anyhow, CHIs should be used more regulated and cautiously, especially in children.

### Strength and Limitation

The major strength of the current study included comprehensive search strategies and analyses. Furthermore, we performed sensitivity analyses to assess the robustness of the results and carried out network meta-regression as well as subgroup analyses to address the heterogeneity of the selected studies. Meanwhile, a 5-dimensional K-means cluster analysis was employed to comprehensively compare the treatment effects of the selected CHIs on the five outcomes. However, some limitations to this study should be mentioned. First, all the outcomes were rated as “high risk” and “some concerns”, for which the results should be interpreted cautiously. Secondary, the WM treatment regimens of the included studies were inconsistent; hence, the results should be interpreted with caution. Third, all the studies were conducted in China and the results may not be generalizable. Finally, several CHIs were associated with small numbers of RCTs (CHN, one RCTs; QKL, three RCTs; SHL, four RCTs; XBJ, three RCTs; YXC, four RCTs) and the interpretation of the results might be restricted.

## Conclusion

CHIs combined with WM have more favorable effects than WM alone in treating acute tonsillitis. QKL, XBJ, RDN, SHL, and YHN deserve more attention when facing patients with acute tonsillitis. Taking ADRs into consideration, XBJ was probably the best CHI for the disease. More evidence, however, is required to support these suggestions.

## Data Availability

The original contributions presented in the study are included in the article/Supplementary Material, further inquiries can be directed to the corresponding author.
