# Supplementary material for: A Five-Dimensional Network Meta-Analysis of Chinese Herbal Injections for Treating Acute Tonsillitis Combined With Western Medicine
Source: Front Pharmacol. 2022 Jun 17;13:888073. doi: 10.3389/fphar.2022.888073 (PMC9247210; doi:10.3389/fphar.2022.888073)
Supplement: Supplementary file 1 [file DataSheet1.docx]

Supplementary Material

Content

[1 File S1: PRISMA checklist for network meta-analysis. 1](#_Toc102118095)

[2 File S2: Search strategy for network meta-analysis. 5](#_Toc102118096)

[2.1 Search strategy of China National Knowledge Infrastructure. 5](#_Toc102118097)

[2.2 Search strategy of Wanfang Database. 5](#_Toc102118098)

[2.3 Search strategy of Chinese Biomedical Literature Database. 5](#_Toc102118099)

[2.4 Search strategy of Weipu Journal Database. 6](#_Toc102118100)

[2.5 Search strategy of Pubmed. 6](#_Toc102118101)

[2.6 Search strategy of Embase. 7](#_Toc102118102)

[2.7 Search strategy of Cochrane Library. 7](#_Toc102118103)

[2.8 Search strategy of Web of Science. 8](#_Toc102118104)

[3 File S3: Citations of the included studies. 9](#_Toc102118105)

[4 File S4: Flow chart for studies screening. 16](#_Toc102118106)

[5 File S5: Details of the included CHIs. 17](#_Toc102118107)

[6 File S6: Characteristics of the included studies. 22](#_Toc102118108)

[7 File S7: Forest plot of inconsistency in clinical effectiveness rate. 35](#_Toc102118109)

[8 File S8: Heatmap for contribution degree of inconsistency in clinical effectiveness rate. 36](#_Toc102118110)

[9 File S9: Results of Egger’s test. 37](#_Toc102118111)

[9.1 Egger’s test for clinical effectiveness rate. 37](#_Toc102118112)

[9.2 Egger’s test for antipyretic time. 37](#_Toc102118113)

[9.3 Egger’s test for sore throat relief time. 37](#_Toc102118114)

[9.4 Egger’s test for red and swollen tonsils relief time. 37](#_Toc102118115)

[9.5 Egger’s test for tonsillar exudate relief time. 37](#_Toc102118116)

[10 File S10: Forest plots of network meta-regression. 39](#_Toc102118117)

[11 File S11: Sensitivity analysis. 40](#_Toc102118118)

[12 File S12: Subgroup of paediatric patients. 42](#_Toc102118119)

[13 File S13: Subgroup of patients with suppurative tonsillitis. 46](#_Toc102118120)

[14 File S14: Subgroup for patients who received penicillins as the treatment regimen of WM. 50](#_Toc102118121)

[15 File S15: Subgroup for patients who received cephalosporins as the treatment regimen of WM. 53](#_Toc102118122)

# File S1: PRISMA checklist for network meta-analysis.

| **Section/topic** | **#** | **Checklist item** | **Reported on page #** |
| --- | --- | --- | --- |
| **TITLE** | | |  |
| Title | 1 | Identify the report as a systematic review incorporating a network meta-analysis (or related form of  meta-analysis). | 1 |
| **ABSTRACT** | | |  |
| Structured summary | 2 | Provide a structured summary including, as applicable:  Background: main objectives  Methods: data sources; study eligibility criteria, participants, and interventions; study appraisal; and synthesis methods, such as network meta-analysis.  Results: number of studies and participants identified; summary estimates with corresponding confidence/credible intervals; treatment rankings may also be discussed. Authors may choose to summarize pairwise comparisons against a chosen treatment included in their analyses for brevity.  Discussion/Conclusions: limitations; conclusions and implications of findings.  Other: primary source of funding; systematic review registration number with registry name. | 1-2, 11 |
| **INTRODUCTION** | | |  |
| Rationale | 3 | Describe the rationale for the review in the context of what is already known, including mention of why a network meta-analysis has been conducted | 2 |
| Objectives | 4 | Provide an explicit statement of questions being addressed with reference to participants, interventions, comparisons, outcomes, and study design (PICOS). | 2 |
| **METHODS** | | |  |
| Protocol and registration | 5 | Indicate if a review protocol exists and if and where it can be accessed (e.g., Web address), and, if available, provide registration information including registration number. | 2 |
| Eligibility criteria | 6 | Specify study characteristics (e.g., PICOS, length of follow-up) and report characteristics (e.g., years considered, language, publication status) used as criteria for eligibility, giving rationale. Clearly describe eligible treatments included in the treatment network and note whether any have been clustered or merged into the same node (with justification). | 2 |
| Information sources | 7 | Describe all information sources (e.g., databases with dates of coverage, contact with study authors to identify additional studies) in the search and date last searched. | 2 |
| Search | 8 | Present full electronic search strategy for at least one database, including any limits used, such that it could be repeated. | 2 |
| Study selection | 9 | State the process for selecting studies (i.e., screening, eligibility, included in systematic review, and, if applicable, included in the meta-analysis). | 2 |
| Data collection process | 10 | Describe method of data extraction from reports (e.g., piloted forms, independently, in duplicate) and any processes for obtaining and confirming data from investigators. | 3 |
| Data items | 11 | List and define all variables for which data were sought (e.g., PICOS, funding sources) and any assumptions and simplifications made. | 3 |
| Geometry of the network | S1 | Describe methods used to explore the geometry of the treatment network under study and potential biases related to it. This should include how the evidence base has been graphically summarized for presentation, and what characteristics were compiled and used to describe the evidence base to readers | 3 |
| Risk of bias within individual studies | 12 | Describe methods used for assessing risk of bias of individual studies (including specification of whether this was done at the study or outcome level), and how this information is to be used in any data synthesis. | 3 |
| Summary measures | 13 | State the principal summary measures (e.g., risk ratio, difference in means). Also describe the use of additional summary measures assessed, such as treatment rankings and surface under the cumulative ranking curve (SUCRA) values, as well as modified approaches used to present summary findings from meta-analyses. | 3 |
| Planned methods of analysis | 14 | Describe the methods of handling data and combining results of studies for each network meta-analysis. This should include, but not be limited to: Handling of multigroup trials; Selection of variance structure; Selection of prior distributions in Bayesian analyses; and Assessment of model fit. | 3 |
| Assessment of inconsistency | S2 | Describe the statistical methods used to evaluate the agreement of direct and indirect evidence in the treatment network(s) studied. Describe efforts taken to address its presence when found. | 3 |
| Risk of bias across studies | 15 | Specify any assessment of risk of bias that may affect the cumulative evidence (e.g., publication bias, selective reporting within studies) | 3 |
| Additional analyses | 16 | Describe methods of additional analyses if done, indicating which were prespecified. This may include, but not be limited to, the following: Sensitivity or subgroup analyses; Meta-regression analyses; Alternative formulations of the treatment network; and Use of alternative prior distributions for Bayesian analyses (if applicable). | 3 |
| **RESULTS** | | |  |
| Study selection | 17 | Give numbers of studies screened, assessed for eligibility, and included in the review, with reasons for exclusions at each stage, ideally with a flow diagram. | 3 |
| Presentation of network structure | S3 | Provide a network graph of the included studies to enable visualization of the geometry of the treatment network. | 3 |
| Summary of network geometry | S4 | Provide a brief overview of characteristics of the treatment network. This may include commentary on the abundance of trials and randomized patients for the different interventions and pairwise comparisons in the network, gaps of evidence in the treatment network, and potential biases reflected by the network structure. | 3 |
| Study characteristics | 18 | For each study, present characteristics for which data were extracted (e.g., study size, PICOS, follow-up period) and provide the citations. | 3 |
| Risk of bias within studies | 19 | Present data on risk of bias of each study and, if available, any outcome level assessment. | 3-4, 6 |
| Results of individual studies | 20 | For all outcomes considered (benefits or harms), present, for each study: 1) simple summary data for each intervention group, and 2) effect estimates and confidence intervals. Modified approaches may be needed to deal with information from larger networks. | 6-7 |
| Synthesis of results | 21 | Present results of each meta-analysis done, including confidence/credible intervals. In larger networks, authors may focus on comparisons versus a particular comparator (e.g., placebo or standard care), with full findings presented in an appendix. League tables and forest plots may be considered to summarize pairwise comparisons. If additional summary measures were explored (such as treatment rankings), these should also be presented. | 6-7 |
| Exploration for inconsistency | S5 | Describe results from investigations of inconsistency. This may include such information as measures of model fit to compare consistency and inconsistency models, P values from statistical tests, or summary of inconsistency estimates from different parts of the treatment network. | 7 |
| Risk of bias across studies | 22 | Present results of any assessment of risk of bias across studies for the evidence base being studied. | 7-8 |
| Results of additional analyses | 23 | Give results of additional analyses, if done (e.g., sensitivity or subgroup analyses, meta-regression  analyses, alternative network geometries studied, alternative choice of prior distributions for  Bayesian analyses, and so forth). | 6-7 |
| **DISCUSSION** | | |  |
| Summary of evidence | 24 | Summarize the main findings, including the strength of evidence for each main outcome; consider their relevance to key groups (e.g., health care providers, researchers, and policymakers). | 8 |
| Limitations | 25 | Discuss limitations at study and outcome level (e.g., risk of bias), and at review level (e.g., incomplete retrieval of identified research, reporting bias). Comment on the validity of the assumptions, such as transitivity and consistency. Comment on any concerns regarding network geometry (e.g., avoidance of certain comparisons). | 11 |
| Conclusions | 26 | Provide a general interpretation of the results in the context of other evidence, and implications for future research. | 11 |
| **FUNDING** | | |  |
| Funding | 27 | Describe sources of funding for the systematic review and other support (e.g., supply of data); role of funders for the systematic review. This should also include information regarding whether funding has been received from manufacturers of treatments in the network and/or whether some of the authors are content experts with professional conflicts of interest that could affect use of treatments in the network. | 11 |

# File S2: Search strategy for network meta-analysis.

## Search strategy of China National Knowledge Infrastructure.

| No. | Search items |
| --- | --- |
| #1 | SU %= '扁桃体炎’ OR SU %= '扁桃腺炎' OR SU %= '乳蛾' OR SU %= '扁桃体周围脓肿' |
| #2 | SU %= '注射剂' OR SU %= '注射液' OR SU %= '痰热清' OR SU %= '喜炎平' OR SU %= '热毒宁' OR SU %= '血必净' OR SU %= '鱼腥草' OR SU %= '炎琥宁' OR SU %= '穿琥宁' OR SU %= '清开灵' OR SU %= '双黄连' OR SU %= '莲必治' OR SU %= '野菊花' OR SU %= '柴胡' |
| #3 | FT = '随机' |
| #4 | #1 AND #2 AND #3 |

## Search strategy of Wanfang Database.

| No. | Search items |
| --- | --- |
| #1 | 主题:(扁桃体炎) or 主题:(扁桃腺炎) or 主题:(乳蛾) or 主题:(扁桃体周围脓肿) |
| #2 | 主题:(注射剂) or 主题:(注射液) or 主题:(痰热清) or 主题:(喜炎平) or 主题:(热毒宁) or 主题:(血必净) or 主题:(鱼腥草) or 主题:(炎琥宁) or 主题:(穿琥宁) or 主题:(清开灵) or 主题:(双黄连) or 主题:(莲必治) or 主题:(野菊花) or 主题:(柴胡) |
| #3 | 全部:(随机) |
| #4 | #1 AND #2 AND #3 |

## Search strategy of Chinese Biomedical Literature Database.

| No. | Search items |
| --- | --- |
| #1 | "扁桃体炎"[常用字段:智能] OR "扁桃腺炎"[常用字段:智能] OR "乳蛾"[常用字段:智能] OR "扁桃体周围脓肿"[常用字段:智能] |
| #2 | "清开灵"[常用字段:智能] OR "双黄连"[常用字段:智能] OR "莲必治"[常用字段:智能] OR "野菊花"[常用字段:智能] OR "柴胡"[常用字段:智能] OR "注射液"[常用字段:智能] OR "注射剂"[常用字段:智能] OR "痰热清"[常用字段:智能] OR "喜炎平"[常用字段:智能] OR "热毒宁"[常用字段:智能] OR "血必净"[常用字段:智能] OR "鱼腥草"[常用字段:智能] OR "炎琥宁"[常用字段:智能] OR "穿琥宁"[常用字段:智能] |
| #3 | "随机"[全部字段:智能]) |
| #4 | #1 AND #2 AND #3 |

## Search strategy of Weipu Journal Database.

| No. | Search items |
| --- | --- |
| #1 | M=(扁桃体炎 OR 扁桃腺炎 OR 乳蛾 OR 扁桃体周围脓肿) |
| #2 | M=(注射剂 OR 注射液 OR 痰热清 OR 喜炎平 OR 热毒宁 OR 血必净 OR 鱼腥草 OR 炎琥宁 OR 穿琥宁 OR 清开灵 OR 双黄连 OR 莲必治 OR 野菊花 OR 柴胡) |
| #3 | U=(随机) |
| #4 | #1 AND #2 AND #3 |

## Search strategy of Pubmed.

| No. | Search items |
| --- | --- |
| #1 | "Tonsillitis"[Mesh] |
| #2 | tonsillitis [Title/Abstract] OR peritonsillar abscess[Title/Abstract] OR abscesses, peritonsillar[Title/Abstract] OR abscess, peritonsillar[Title/Abstract] OR (tonsil*[Title/Abstract] AND inflam*[Title/Abstract]) OR (tonsil*[Title/Abstract] AND infect*[Title/Abstract]) |
| #3 | #1 OR #2 |
| #4 | Chinese Herbal Injection[Title/Abstract] OR Traditional Chinese Medicine Injection[Title/Abstract] OR tanreqing[Title/Abstract] OR xiyanping[Title/Abstract] OR reduning[Title/Abstract] OR xuebijing[Title/Abstract] OR yuxingcao[Title/Abstract] OR yanhuning[Title/Abstract] OR chuanhuning[Title/Abstract] OR qingkailing[Title/Abstract] OR shuanghuanglian[Title/Abstract] OR lianbizhi[Title/Abstract] OR yejuhua[Title/Abstract] OR chaihu[Title/Abstract] |
| #5 | Controlled Clinical Trial [Publication Type] OR Randomized Controlled Trial[Publication Type] OR Equivalence Trial[Publication Type] OR Pragmatic Clinical Trial[Publication Type] OR random*[All Fields] |
| #6 | #3 AND #4 AND #5 |

## Search strategy of Embase.

| No. | Search items |
| --- | --- |
| #1 | 'tonsillitis'/exp OR 'tonsillitis':ti,ab,kw OR 'peritonsillar abscess':ti,ab,kw OR 'abscesses, peritonsillar':ti,ab,kw OR 'abscess, peritonsillar':ti,ab,kw OR (tonsil*:ti,ab,kw AND inflam*:ti,ab,kw) OR (tonsil*:ti,ab,kw AND infect*:ti,ab,kw) |
| #2 | 'chinese herbal injection':ti,ab,kw OR 'traditional chinese medicine injection':ti,ab,kw OR tanreqing:ti,ab,kw OR xiyanping:ti,ab,kw OR reduning:ti,ab,kw OR xuebijing:ti,ab,kw OR yuxingcao:ti,ab,kw OR yanhuning:ti,ab,kw OR chuanhuning:ti,ab,kw OR qingkailing:ti,ab,kw OR shuanghuanglian:ti,ab,kw OR lianbizhi:ti,ab,kw OR yejuhua:ti,ab,kw OR chaihu:ti,ab,kw |
| #3 | 'randomized controlled trial'/exp OR 'equivalence trial'/exp OR 'non-inferiority trial'/exp OR 'pragmatic trial'/exp OR 'superiority trial'/exp OR 'controlled clinical trial':it OR 'randomized controlled trial':it OR 'equivalence trial':it OR 'pragmatic clinical trial':it OR 'superiority trial':it OR 'non-inferiority trial':it OR random* |
| #4 | #1 AND #2 AND #3 |

## Search strategy of Cochrane Library.

| No. | Search items |
| --- | --- |
| #1 | MeSH descriptor: [Tonsillitis] explode all trees |
| #2 | (tonsillitis):ti,ab,kw OR (peritonsillar abscess):ti,ab,kw OR (abscesses, peritonsillar):ti,ab,kw OR (abscess, peritonsillar):ti,ab,kw OR ((tonsil*):ti,ab,kw AND (inflam*):ti,ab,kw) OR ((tonsil*):ti,ab,kw AND (infect*):ti,ab,kw) |
| #3 | #1 OR #2 |
| #4 | (Chinese Herbal Injection):ti,ab,kw OR (Traditional Chinese Medicine Injection):ti,ab,kw OR (tanreqing):ti,ab,kw OR (xiyanping):ti,ab,kw OR (reduning):ti,ab,kw OR (xuebijing):ti,ab,kw OR (yuxingcao):ti,ab,kw OR (yanhuning):ti,ab,kw OR (chuanhuning):ti,ab,kw OR (qingkailing):ti,ab,kw OR (shuanghuanglian):ti,ab,kw OR (lianbizhi):ti,ab,kw OR (yejuhua):ti,ab,kw OR (chaihu):ti,ab,kw |
| #5 | MeSH descriptor: [Randomized Controlled Trial] explode all trees |
| #6 | (Randomized Controlled Trial):pt OR (Controlled Clinical Trial):pt OR (Equivalence Trial):pt OR (Pragmatic Clinical Trial):pt OR (random*) |
| #7 | #5 OR #6 |
| #8 | #3 AND #4 AND #7 |

## Search strategy of Web of Science.

| No. | Search items |
| --- | --- |
| #1 | (tonsillitis (Topic) or peritonsillar abscess (Topic) or abscesses, peritonsillar (Topic) or abscess, peritonsillar (Topic) or (tonsil* (Topic) and inflam* (Topic)) or (tonsil* (Topic) and infect* (Topic)) |
| #2 | (chinese herbal injection (Topic) or traditional chinese medicine injection (Topic) or tanreqing (Topic) or xiyanping (Topic) or reduning (Topic) or xuebijing (Topic) or yuxingcao (Topic) or yanhuning (Topic) or chuanhuning (Topic) or qingkailing (Topic) or shuanghuanglian (Topic) or lianbizhi (Topic) or yejuhua (Topic) or chaihu (Topic) |
| #3 | random* (All Fields) |
| #4 | #1 AND #2 AND #3 |

# File S3: Citations of the included studies.

Bie, X. (2018). Observation of curative effect of Reduning in children with suppurative tonsillitis. *Women's Health Research* (5). doi: 10.3969/j.issn.2096-0417.2018.05.006.

Cai, B. (2013). Clinical observation of Tanreqing injection in the treatment of acute suppurative tonsillitis. *Modern Diagnosis & Treatment* 24(4). doi: 10.3969/j.issn.1001-8174.2013.04.046.

Cai, Y., Chen, J., and Luo, X. (2011). Analysis of the effect of Xuebijing on 60 cases of acute suppurative tonsillitis. *CHINESE JOURNAL OF COAL INDUSTRY MEDICINE* 14(08)**,** 1183. doi: CNKI:SUN:ZMGY.0.2011-08-049.

Che, Y., and Xu, Y. (2006). Observation on the effect of Tanreqing injection in treating 49 cases of infantile suppurative tonsillitis. *Chinese Journal of Clinical Medicine* 7(2).

Chen, W. (2014). Clinical effect of Reduning on 32 cases of suppurative tonsillitis. *For All Health* (10)**,** 282-282. doi: CNKI:SUN:JKXS.0.2014-19-361.

Chen, X. (2009). Clinical observation of Reduning combined with cefuroxime in the treatment of 30 cases of suppurative tonsillitis. *GUANGXI MEDICAL JOURNAL* 31(05)**,** 711-712. doi: 10.3969/j.issn.0253-4304.2009.05.056.

Chen, Y. (2020). Reduning combined with cefuroxime in the treatment of acute suppurative tonsillitis. *Diet Health* 7(13)**,** 80.

Deng, W. (2015). Observation on the curative effect of Tanreqing injection combined with ceftizole sodium on acute tonsillitis. *Anti-Infection Pharmacy* 12(3). doi: 10.13493/j.issn.1672-7878.2015.03-056.

Ding, J., and Luo, l. (2010). Observation of Curative Effect of Reduning on Infantile Suppurative Tonsillitis. *ACTA CHINESE MEDICINE AND PHARMACOLOGY* 38(6). doi: 10.3969/j.issn.1002-2392.2010.06.022.

Gao, L., and Xu, C. (2006). Houttuynia cordata injection in the treatment of 34 children with acute suppurative tonsillitis. *ZHEJIANG JOURNAL OF TRADITIONAL CHINESE MEDICINE* (04)**,** 238. doi: 10.3969/j.issn.0411-8421.2006.04.041.

Gao, Y. (2012). Observation of the curative effect of Yanhuning on acute suppurative tonsillitis in children. *CHINESE JOURNAL OF CLINICAL RATIONAL DRUG USE* 5(24)**,** 135. doi: 10.3969/j.issn.1674-3296.2012.24.108.

Ge, F. (2014). Analysis of clinical efficacy of Reduning injection in the treatment of acute pharyngeal tonsillitis. *Chinese Journal of Clinical Rational Drug Use* (36)**,** 137-138. doi: 10.3969/j.issn.1674-3296.2014.36.096.

Ge, L., and Wu, X. (2015). Clinical analysis of Reduning injection in the treatment of children with purulent tonsillitis. *Medical Information* (24)**,** 334-334,335. doi: 10.3969/j.issn.1006-1959.2015.24.517.

Ge, W., and Chen, W. (2012). Clinical observation of Yanhuning injection in treating 115 children with acute suppurative tonsillitis. *Yiayao Qianyan* (25)**,** 216.

Gong, T. (2020). Clinical effect of Xiyanping Injection in the treatment of children with acute suppurative tonsillitis. *Chinese Community Doctors* 36(7). doi: 10.3969/j.issn.1007-614x.2020.07.066.

Gu, J. (2019). Clinical study of Xiyanping injection in the treatment of acute suppurative tonsillitis in children. *Psychological Monthly* 14(05)**,** 168. doi: 10.19738/j.cnki.psy.2019.05.162.

Guo , C. (2013). Clinical Observation of Comprehensive Therapy in Treating Acute Tonsillitis. *CHINESE COMMUNITY DOCTORS* 15(05)**,** 139. doi: 10.3969/j.issn.1007-614x.2013.05.130.

Guo, X., and Ruan, Y. (2001). Observation of Houttuynia cordata combined with penicillin in the treatment of suppurative tonsillitis. *MODERN JOURNAL OF INTEGRATED CHINESE TRADITIONAL AND WESTERN MEDICINE* (24)**,** 2366-2367. doi: 10.3969/j.issn.1008-8849.2001.24.029.

He, M. (2001). Curative effect observation of Chuanhuning combined with cefazolin sodium in the treatment of 48 children with acute tonsillitis. *CHINESE GENERAL PRACTICE* (07)**,** 575. doi: 10.3969/j.issn.1007-9572.2001.07.045.

He, Q. (2015). Exploration of clinical curative effect of Tanreqing injection in the treatment of acute suppurative tonsillitis in children. *Guide of China Medicine* 13(20). doi: CNKI:SUN:YYXK.0.2015-20-178.

He, Q., and Fang, M. (2015). Clinical observation on adjuvant treatment of children with suppurative tonsillitis by traditional Chinese medicine Reduning. *Journal of New Chinese Medicine* 47(7). doi: 10.13457/j.cnki.jncm.2015.07.090.

He, X. (2013). Efficacy observation of Xiyanping combined with clindamycin in the treatment of acute suppurative tonsillitis in children. *China Health Industry* 10(32).

Hu, Q., Zhang, L., Xu, X., Ouyang, S., Yao, R., and Wang, X. (2017). Clinical observation of Tanreqing injection combined with antibiotics in the treatment of acute suppurative tonsillitis in children. *Journal of North Pharmacy* 14(2). doi: 10.3969/j.issn.1672-8351.2017.02.104.

Huang, C., Li, Z., and Tang, X. (2014). Clinical observation of Tanreqing injection combined with cefathiamidine in the treatment of acute suppurative tonsillitis. *Journal of Chinese Medicinal Materials* (6).

Huang, T. (2018). To observe the clinical efficacy of Tanreqing injection combined with cefathiamidine in the treatment of acute suppurative tonsillitis. *Bao Jian Wen Hui* (6)**,** 8. doi: 10.3969/j.issn.1671-5217.2018.06.008.

Jiang, J., and Yang, T. (2012). Clinical observation of Xiyanping injection and antibiotic treatment of acute suppurative tonsillitis. *CHINESE JOURNAL OF CLINICAL RATIONAL DRUG USE* 5(8). doi: 10.3969/j.issn.1674-3296.2012.08.030.

Kuai, H. (2013). 26 cases of acute suppurative tonsillitis treated with Reduning combined with ceftizoxime. *CHINESE MEDICINE MODERN DISTANCE EDUCATION OF CHINA* (19). doi: 10.3969/j.issn.1672-2779.2013.19.047.

Li, A. (2019). Clinical observation of Reduning adjuvant treatment of children with suppurative tonsillitis. *Journal of Clinical Rational Drug Use* (1). doi: CNKI:SUN:PLHY.0.2019-01-070.

Li, F. (2018). Study on the value of Yanhuning in the treatment of acute suppurative tonsillitis in children. *Cardiovascular Disease Journal of Integrated Traditional Chinese and Western Medicine(Electronic)* 6(01)**,** 87. doi: 10.16282/j.cnki.cn11-9336/r.2018.01.063.

li, H., Zhang, J., Cui, Z., and Wang, Y. (2020a). The value of serum inflammatory factors IL-6 and TNF-α levels in the adjuvant treatment of acute suppurative tonsillitis with Tanreqing injection. *Diet Health* 7(13)**,** 42.

Li, H., Zhang, X., Fan, S., Liu , C., and Ren, S. (2020b). The role and significance of TLR4/NF-κB signaling pathway-mediated inflammato-ry response in the treatment of acute tonsillitis. *Northwest Pharmaceutical Journal* 35(5). doi: 10.3969/j.issn.1004-2407.2020.05.018.

li, J. (2017). Observation on the curative effect of cefuroxime sodium in the treatment of children with suppurative tonsillitis. *Health Guide* (43)**,** 234. doi: 10.3969/j.issn.1006-6845.2017.43.222.

Li, J., and Li , L. (2004). Observation on the curative effect of Xiyanping in the treatment of children with suppurative tonsillitis. *JOURNAL OF LIAONING COLLEGE OF TRADITIONAL CHINESE MEDICINE* (04)**,** 318. doi: 10.3969/j.issn.1673-842X.2004.04.038.

Li , Q. (2013). 18 cases of suppurative tonsillitis treated with Reduning. *CHINESE MEDICINE MODERN DISTANCE EDUCATION OF CHINA* 11(23)**,** 43-43. doi: 10.3969/j.issn.1672-2779.2013.23.028.

Li , X., and Wang, G. (2015). 100 cases of acute pharyngeal tonsillitis infection treated by Reduning injection. *Lishizhen Medicine and Materia Medica Research* 26(04)**,** 926. doi: 10.3969/j.issn.1008-0805.2015.04.065.

Li, Z. (2012). Efficacy observation of treating 30 cases of children with acute purulent tonsillitis with antibiotics joint Xue Bi Jing injection. *Clinical Journal of Chinese Medicine* 4(13). doi: 10.3969/j.issn.1674-7860.2012.13.054.

Lin , S., Qu, J., and Wang, X. (2002). Clinical observation of Qingkailing injection in the treatment of acute suppurative tonsillitis. *HEILONGJIANG JOURNAL OF TRADITIONAL CHINESE MEDICINE* (03)**,** 35. doi: CNKI:SUN:HLZY.0.2002-03-023.

Liu, H. (2014). Clinical Study on Reduning Treating 56 Cases of Acute Suppurative Tonsillitis. *For All Health* 8(02)**,** 60. doi: 10.3969/j.issn.1009-6019.2014.01.067.

Liu, P., Song, D., and Yang, S. (2008). Observation on the effect of Xiyanping in the treatment of acute suppurative tonsillitis in children. *CHINA PRACTICAL MEDICINE* (17)**,** 109-110. doi: 10.3969/j.issn.1673-7555.2008.17.091.

Liu, Y. (2020). Efficacy evaluation of Xiyanping injection in the treatment of children with tonsillitis. *Heilongjiang Medicine Journal* 33(5). doi: 10.14035/j.cnki.hljyy.2020.05.030.

Long, D. (2015). Shuanghuanglian Injection Combined with Penicillin Treatment of Infantile Suppurative Tonsillitis Observation. *Modern Diagnosis & Treatment* 26(19)**,** 4369-4371. doi: CNKI:SUN:XDZD.0.2015-19-032.

Long, Q., and Cai, X. (2014). Clinical study of Xiyanping injection combined with mezlocillin and sulbactam sodium in the treatment of acute suppurative tonsillitis in children. *Chinese Journal of Control of Endemic Diseases* 29(S2)**,** 234-235. doi: CNKI:SUN:DYBF.0.2014-S2-266.

Lu, S. (2010). Clinical observation of amoxicillin-clavulanate potassium combined with Xiyanping in the treatment of acute suppurative tonsillitis in children. *CHINESE JOURNAL OF MODERN DRUG APPLICATION* (24). doi: 10.3969/j.issn.1673-9523.2010.24.130.

Luo, L. (2003). Observation of curative effect of Houttuynia cordata injection in the treatment of acute suppurative tonsillitis. *CHONGQING MEDICAL JOURNAL* (11)**,** 1501. doi: 10.3969/j.issn.1671-8348.2003.11.079.

Luo, W. (2016). Clinical observation of clindamycin combined with Xiyanping in the treatment of acute suppurative tonsillitis in children. *Psychological Doctor* 22(27)**,** 145-146.

Lv, Y. (2020). Clinical study of Xiyanping injection in treating acute suppurative tonsillitis in children. *Healthful Friend* (18)**,** 286.

Ma, J. (2015). Observation on the therapeutic effect of Xiyanping combined with azithromycin in 48 children with acute tonsillitis. *Journal of North Pharmacy* 12(12)**,** 63. doi: CNKI:SUN:BFYX.0.2015-12-055.

Ma, N., and Wang, H. (2013). Clinical study of Reduning combined with penicillin in the treatment of acute suppurative tonsillitis in children. *Chinese Community Doctors* 15(21)**,** 67. doi: CNKI:SUN:ZGSQ.0.2013-21-055.

Niu, Z. (2005). Tanreqing injection combined with penicillin injection in the treatment of 38 cases of acute suppurative tonsillitis. *TRADITIONAL CHINESE MEDICINAL RESEARCH* (05)**,** 45-46. doi: 10.3969/j.issn.1001-6910.2005.05.024.

Ouyang, X., Liu, H., and Xu, H. (2017). Clinical Study of Xiyanping Injection for Treatment of Children with Acute Suppurative Tonsillitis. *Journal of Guangzhou University of Traditional Chinese Medicine* 34(4). doi: 10.13359/j.cnki.gzxbtcm.2017.04.006.

Pan, L., Shen, N., Zhang, Y., Zhang, X., Li, L., Liu , L., et al. (2014a). Efficacy observation of Xiyanping combined with azithromycin in the treatment of acute tonsillitis in children. *Hebei Medical Journal* 36(10)**,** 1537-1538. doi: 10.3969/j.issn.1002-7386.2014.10.045.

Pan, L., Zhang, Y., Zhang, X., Xiong, N., Du, W., and Li , L. (2014b). Curative effect observation of Xiyanping and Tanreqing Injection in the treatment of children with acute suppurative tonsillitis. *Journal of Pediatrics of Traditional Chinese Medicine* 10(06)**,** 19-21. doi: CNKI:SUN:ZYEZ.0.2014-06-011.

Peng, X., Chen, L., and Wang, J. (2015). Effect of clindamycin alone or combined with Xiyanping injection on acute suppurative tonsillitis in children. *Chinese Archives of Otolaryngology-Head and Neck Surgery* 22(04)**,** 204-206. doi: 10.16066/j.1672-7002.2015.04.013.

Qiao, Y., Yu, X., Deng, J., Qiu, Y., Shu, H., and Luo, Y. (2015). Clinical Efficacy of Xiyanping Injection Combined withβ-lactam Antibiotics inTreatment of Children with Acute Suppurative Tonsillitis. *Journal of Modern Clinical Medicine* (4)**,** 258-259,262. doi: 10.11851/j.issn.1673-1557.2015.04.006.

Qiu, L. (2019). Observation on the curative effect of Reduning in the treatment of children with suppurative tonsillitis. *Guide of China Medicine* 17(10). doi: CNKI:SUN:YYXK.0.2019-10-154.

Ren, L. (2016). To explore the efficacy of Reduning injection combined with cefuroxime in the treatment of children with suppurative tonsillitis. *Clinical Research* 24(6).

Ren, X. (2018). Observation on the effect of Xiyanping and clindamycin in the treatment of children with acute suppurative tonsillitis. *Gansu Science and Technology* 34(2). doi: 10.3969/j.issn.1000-0952.2018.02.046.

Shen, Y., and Duan, X. (Year). "Efficacy observation of Reduning injection combined with mezlocillin and sulbactam sodium in the treatment of acute suppurative tonsillitis", in: *Symposium on Translational Medicine and Integrative Medicine in China*), 138-139.

Shi, L. (2018). Clinical study of Xiyanping injection in treating acute suppurative tonsillitis in children. *Psychological Doctor* 24(7)**,** 75.

Shi, Y., and Lian, Q. (2006). Clinical Observation on Acute Tonsillitis Treated by Integrated Traditional Chinese and Western Medicine. *CHINA MEDICAL HERALD* (24)**,** 116. doi: 10.3969/j.issn.1673-7210.2006.24.088.

Shuai, C., Liang, Q., and Liu, H. (2018). Clinical study of Xiyanping injection in the treatment of acute suppurative tonsillitis in children. *Contemporary Medicine* 24(23). doi: 10.3969/j.issn.1009-4393.2018.23.044.

Song, H., Ma, L., and Yue, H. (2020). Effects of Reduning Injection Combined with Conventional Western Medicine on Inflammatory Factors and Immune Function in Patients with Acute Suppurative Tonsillitis. *Research of Integrated Traditional Chinese and Western Medicine* 12(03)**,** 149-153. doi: 10.3969/j.issn.1674-4616.2020.03.002.

Song, Y., Wang, J., Feng, D., Hua, L., and Zhang, Y. (2018). Effect of adjuvant therapy with XiYanPing on the acute suppurative tonsillutis in children. *Journal of Clinical Medical Literature （ElectronicEdition）* 5(29)**,** 8-9. doi: 10.3877/j.issn.2095-8242.2018.29.005.

Su, P. (2015). Clinical observation of Yanhuning in the treatment of acute suppurative tonsillitis in children. *Contemporary Medicine* 21(34). doi: 10.3969/j.issn.1009-4393.2015.34.085.

Sun, T. (2020). Effects of cefprozil combined with Xiyanping on the levels of PCT, TNF-α and IL-6 in the treatment of child patients with acute suppurative tonsillitis. *China Practical Medical* 15(21)**,** 22-24. doi: 10.14163/j.cnki.11-5547/r.2020.21.008.

Tang, Y., and Du, Z. (2008). Observation on the curative effect of Tanreqing injection in the treatment of suppurative tonsillitis. *JOURNAL OF EMERGENCY IN TRADITIONAL CHINESE MEDICINE* (04)**,** 457. doi: 10.3969/j.issn.1004-745X.2008.04.019.

Wang, H. (2015). Efficacy evaluation of Xiyanping combined with clindamycin in the treatment of children with acute suppurative tonsillitis. *Henan Medical Research* (10)**,** 91-92. doi: 10.3969/j.issn.1004-437X.2015.10.050.

Wang, J. (2016). Observation on the effect of Xiyanping combined with mezlocillin sodium in the treatment of acute tonsillitis. *China Health Care & Nutrition* 26(27)**,** 303.

Wang, W. (2018). Observation on the curative effect of Xiyanping injection in the treatment of acute tonsillitis. *Modern Medicine and Health Research* 0(13). doi: CNKI:SUN:XYJD.0.2018-13-098.

Wang, Y. (2014). Clinical observation on 125 cases of acute suppurative tonsillitis treated with mezlocillin and sulbactam sodium combined with Reduning. *Asia-Pacific Traditional Medicine* 10(18).

Wang, Y. (2020). Clinical effect analysis of Xiyanping injection in the treatment of children with acute suppurative tonsillitis. *Journal of Clinical Medical Literature （ElectronicEdition）* 7(A1)**,** 163-164.

Wang, Y., and Lu, G. (2015). Clinical observation of Reduning combined with azithromycin in the treatment of 128 cases of children with suppurative tonsillitis. *Guiding Journal of Traditional Chinese Medicine and Pharmacology* 21(23)**,** 74-76. doi: CNKI:SUN:HNZB.0.2015-23-027.

Wei, R. (1998). Qingkailing injection in the treatment of 50 cases of infantile tonsillitis. *Medical Theory and Practice* (10)**,** 469-470. doi: 10.19381/j.issn.1001-7585.1998.10.035.

Wu, B. (2017). Clinical observation of effects of Reduning injection on children with acute suppurative tonsillitis. *Medical Journal of Chinese People's Health* 29(8). doi: 10.3969/j.issn.1672-0369.2017.08.020.

Wu, X. (2013). Clinical observation of Xiyanping injection combined with mezlocillin and sulbactam sodium in the treatment of acute suppurative tonsillitis in children. *CHINA HEALTH VISION* 21(2)**,** 514-515.

Xie, B., and Huang, X. (2013). Observation on Clinical Application of Tanreqing Injection in Treating Suppurative Tonsillitis in Emergency Department. *World Chinese Medicine* 8(10)**,** 1197-1198+1201. doi: 10.3969/j.issn.1673-7202.2013.10.022.

Xin, L., Xin, W., Zhang, J., Liu, X., and Wang, Y. (2021). Clinical study of Xiyanping Injection combined with cefoxitin in treatment of acute tonsillitis. *Drugs & Clinic* 36(7)**,** 1458-1461. doi: 10.7501/j.issn.1674-5515.2021.07.024.

Xu, J., Xu, Z., Zhang, Y., and Dong, S. (2017). Efficacy of Reduning injection in the treatment of acute suppurative tonsillitis in children. *Jiangsu Medical Journal* 43(23)**,** 1747-1748. doi: 10.19460/j.cnki.0253-3685.2017.23.029.

Yang, B., and Wang, H. (2010). Clinical observation of Tanreqing injection in the treatment of acute suppurative tonsillitis in children. *CHINA MODERN DOCTOR* 48(30)**,** 123,125. doi: 10.3969/j.issn.1673-9701.2010.30.071.

Yang, B., and Xiao, D. (2015). Efficacy observation of Xiyanping combined with azithromycin in the treatment of acute tonsillitis in children. *Journal of North Pharmacy* 12(07)**,** 41-42. doi: CNKI:SUN:BFYX.0.2015-07-033.

Yang, L., Xie, Y., and He, M. (2012). Clinical study of Reduning combined with antibiotics and nursing guidance in the treatment of 64 cases of acute suppurative tonsillitis. *Chinese Medical Science* 2(15)**,** 89-90. doi: CNKI:SUN:GYKX.0.2012-15-048.

Yang, W. (2009). Clinical observation of Tanreqing injection combined with cefotaxime sodium in the treatment of 30 cases of acute suppurative tonsillitis. *JOURNAL OF EMERGENCY IN TRADITIONAL CHINESE MEDICINE* 18(7)**,** 1076-1076,1132. doi: 10.3969/j.issn.1004-745X.2009.07.029.

Yang, Y., Zhao, H., and Cao, M. (2008). Observation on the effect of Yanhuning in the treatment of acute suppurative tonsillitis in children. *CHINA PRACTICAL MEDICAL* (16)**,** 122. doi: 10.3969/j.issn.1673-7555.2008.16.083.

Yu, C., and Wang, L. (2016). Clinical efficacy of Xuebijing injection in the treatment of suppurative tonsillitis and its effect on serum inflammatory factors. *Journal of Modern Integrative Medicine* 25(23)**,** 2590-2592. doi: 10.3969/j.issn.1008-8849.2016.23.029.

Yu, L. (2015). Clinical observation of Tanreqing injection combined with azithromycin in the treatment of acute suppurative tonsillitis in children. *Journal of Community Medicine* 0(14). doi: CNKI:SUN:SQYX.0.2015-14-021.

Yu, Z., Tang, H., and Wang, L. (2011). Clinical observation on Tanreqing injection combined with ceftezole in the treatment of acute tonsillitis. *CHINESE JOURNAL OF BIOCHEMICAL PHARMACEUTICS* 32(02)**,** 149-150. doi: CNKI:SUN:SHYW.0.2011-02-026.

Yuan, F., and Hou, J. (2018). Clinical observation of Reduning combined with cefathiamidine in the treatment of acute suppurative tonsillitis in children. *China Health Vision* (16)**,** 89. doi: 10.3969/j.issn.1005-0019.2018.16.126.

Yuan, X. (2000). Therapeutic effect of bicoptis root on acute suppurative tonsillitis. *CHINESE JOURNAL OF CONTEMPORARY PEDIATRICS* 2(2). doi: 10.3969/j.issn.1008-8830.2000.02.027.

Zeng, G. (2018). Clinical analysis of Xiyanping injection in the treatment of acute suppurative tonsillitis in children. *Journal of North Pharmacy* 15(8). doi: 10.3969/j.issn.1672-8351.2018.08.039.

Zeng, J. (2012). Clinical observation of Reduning injection combined with mezlocillin and sulbactam sodium in the treatment of 92 cases of acute suppurative tonsillitis. *GUIDE OF CHINA MEDICINE* 10(17). doi: 10.3969/j.issn.1671-8194.2012.17.220.

Zhang, G., Miao, Y., and Zhu, Y. (2013). 60 Cases of Acute Tonsillitis Treated by Integrated Traditional Chinese and Western Medicine. *Global Traditional Chinese Medicine* (z2)**,** 104-104. doi: 10.3969/j.issn.1674-1749.2013.z2.100.

Zhang, H. (2013). Efficacy of Yanhuning combined with antibiotics in the treatment of acute suppurative tonsillitis in children. *Yiayao Qianyan* (1)**,** 176. doi: 10.3969/j.issn.2095-1752.2013.01.187.

Zhang, L. (2005). Efficacy analysis of Tanreqing injection combined with penicillin in the treatment of suppurative tonsillitis. *Chinese Journal of Modern Practical Medicine* 4(12).

Zhang, L. (2007). Observation of curative effect of Reduning on acute tonsillitis complicated with fever in children. *CHINA MEDICAL HERALD* (15)**,** 126. doi: 10.3969/j.issn.1673-7210.2007.15.095.

Zhang, W. (2016). Curative effect observation of Reduning combined with amoxicillin and clavulanate potassium in the treatment of 100 cases of suppurative tonsillitis. *Shenzhen Journal of Integrated Traditional Chinese and Western Medicine* 0(2). doi: 10.16458/j.cnki.1007-0893.2016.02.019.

Zhang, X. (2017). Clinical effect of azithromycin combined with Xiyanping in the treatment of acute suppurative tonsillitis in children. *The World Clinical Medicine* 11(1)**,** 154,157.

Zhang, Z., Qin, Y., Yun, M., Zhao, S., and Wang, H. (2021). Clinical observation of Tanreqing injection in adjuvant treatment of acute tonsillitis in children. *China's Naturopathy* 29(3). doi: 10.19621/j.cnki.11-3555/r.2021.0332.

Zhao, C. (2014). Efficacy analysis of Reduning injection combined with azithromycin in the treatment of 100 cases of acute suppurative tonsillitis. *For All Health* 8(15)**,** 292. doi: 10.3969/j.issn.1009-6019(z).2014.08.380.

Zhao, M., and Liang, L. (2014). Observation on the therapeutic effect of Shuanghuanglian powder injection combined with penicillin in the treatment of children with suppurative tonsillitis. *Asia-Pacific Traditional Medicine* 10(20)**,** 114-115.

Zhao, Q. (2018). Clinical observation of Tanreqing injection combined with penicillin in the treatment of acute suppurative tonsillitis in children. *Modern Medical Imageology* 27(7). doi: CNKI:SUN:XDYY.0.2018-07-111.

Zhao, X. (2015). Discussion on the treatment and nursing of children with suppurative tonsillitis. *Yiayao Qianyan* (22). doi: 10.3969/j.issn.2095-1752.2015.22.259.

Zhao, Y. (2006). Observation on the effect of Qingkailing on acute suppurative tonsillitis. *OCCUPATION AND HEALTH* (21)**,** 1866. doi: 10.3969/j.issn.1004-1257.2006.21.070.

Zhou, H., and Tao, M. (2003). Houttuynia cordata injection in the treatment of 188 cases of acute suppurative tonsillitis. *ZHEJIANG CLINICAL MEDICAL JOURNAL* (08)**,** 600. doi: 10.3969/j.issn.1008-7664.2003.08.031.

Zhou, K. (2018). Analysis of the effect of Xiyanping injection on acute suppurative tonsillitis in children. *China Practical Medical* 13(01)**,** 117-119. doi: 10.14163/j.cnki.11-5547/r.2018.01.068.

Zhou, S., and Bai, M. (2013). Observation on the curative effect of Reduning in the treatment of children with suppurative tonsillitis. *CHINESE JOURNAL OF CLINICAL RATIONAL DRUG USE* 6(20)**,** 69. doi: 10.15887/j.cnki.13-1389/r.2013.20.142.

Zhou, X. (2019). Clinical efficacy of Xiyanping injection in the treatment of children with acute suppurative tonsillitis and observation of relevant time indicators. *Psychological Doctor* 25(5)**,** 68-69.

Zhu, H. (2017). Clinical effect of Tanreqing injection on patients with acute suppurative tonsillitis. *Chinese Journal of Woman and Child Health Research* 28(S4)**,** 59. doi: CNKI:SUN:SANE.0.2017-S4-068.

Zhu, X., Jiang, X., and Yang, X. (2014). Observation on the curative effect of Reduning injection in the treatment of 89 children with acute tonsillitis. *Zhejiang Journal of Traditional Chinese Medicine* 49(06)**,** 437. doi: 10.13633/j.cnki.zjtcm.2014.06.026.

Zou, A. (2010). Observation on the curative effect of integrated traditional Chinese and western medicine in the treatment of acute suppurative tonsillitis in children. *PRACTICAL CLINICAL JOURNAL OF INTEGRATED TRADITIONAL CHINESE AND WESTERN MEDICINE* 10(03)**,** 49. doi: 10.3969/j.issn.1671-4040.2010.03.038.

Zou, R., and Deng, S. (2011). Clinical observation of Yanhuning powder injection in adjuvant treatment of acute suppurative tonsillitis in children. *JILIN MEDICAL JOURNAL* 32(22)**,** 4605-4606. doi: 10.3969/j.issn.1004-0412.2011.22.057.

# File S4: Flow chart for studies screening.


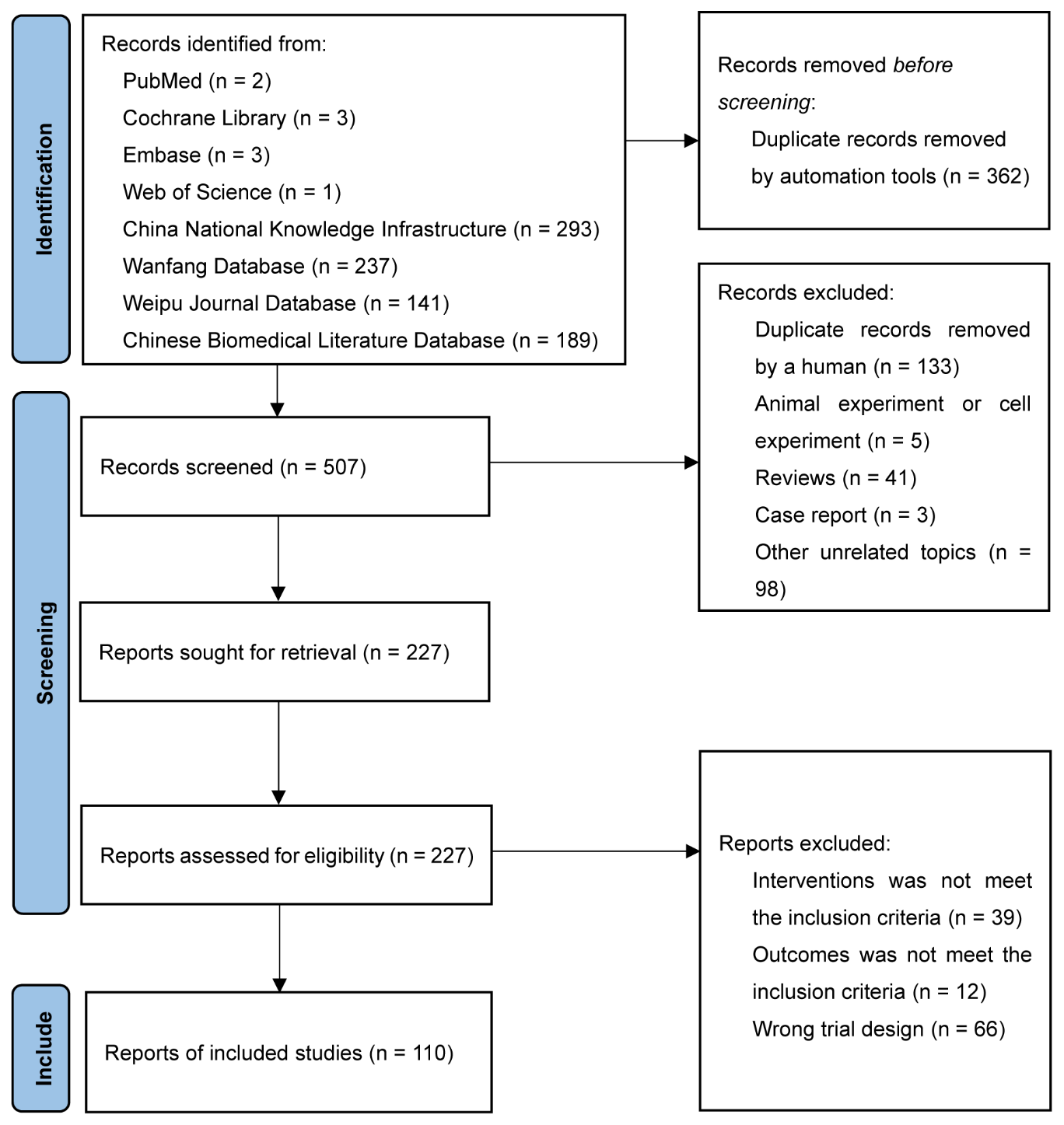


# File S5: Details of the included CHIs.

| **Chinese herbal injections** | **Source** | **Species /Raw materials** | **Scientific name of the plant** | **Indication** | **Quality control reported. (Y/N)** | **Chemical analysis reported (Y/N)** |
| --- | --- | --- | --- | --- | --- | --- |
| Reduning injection | Jiangsu Kangyuan Pharmaceutical Co., Ltd. | Sweet Wormwood Herb, Japanese Honysuckle Flower Bud, Common Gardenia Fruit | *Artemisia annua* L. [Asteraceae],  *Lonicera japonica* Thunb. [Caprifoliaceae], *Gardenia jasminoides* J.Ellis [Rubiaceae] | Cold and cough due to exogenous wind-heat; upper respiratory tract infection; acute bronchitis | Y-National Pharmaceutical Standard: Z20050217; National Food and Drug Administration National Drug Standard: YBZ08202005-2015Z | N |
| Tanreqing injection | Shanghai Kaibao Pharmaceutical Co., Ltd. | Baikal Skullcap Root, Japanese Honysuckle Flower Bud, Weeping Forsythia Fruit, Goral Horn (animal drug), bear bile powder (animal drug) | *Scutellaria baicalensis* Georgi [Lamiaceae],  *Lonicera japonica* Thunb. [Caprifoliaceae],  *Forsythia suspensa* (Thunb.) Vahl [Oleaceae], Ovis ammon Linnaeus, Fel Ursi Selenarctos thibetanus G. Cuvier Ursus arctos L. | Wind-warmth lung heat disease with characteristic of phlegm-heat obstructing lung; early stage of pneumonia; acute bronchitis; acute exacerbation of chronic bronchitis; upper respiratory tract infection | Y-National Pharmaceutical Standard: Z20030054; National Food and Drug Administration National Drug Standard: YBZ00912003-2007Z-2009-2012 | N |
| Yanhuning injection | Hainan Tongyongkangli  Pharmaceutical Co., Ltd.; Chongqing Lummy Pharmaceutical Co.,Ltd.; Fujian Mindong Rejuvenation Pharmaceutic al  Co., Ltd.; Ruikang Pharmaceutical Group Co., Ltd.; Wuhan Renfu Pharmaceutical Co., Ltd.; Beijing Shuanglu Pharmaceutical Co., Ltd. | Andrographolide succinate half ester potassium sodium salt. | *Andrographis paniculata* (Burm.f.) Nees [Acanthaceae] | Viral pneumonia; viral upper respiratory tract infection | Y-National Pharmaceutical Standard: H20055351, H20066107, H20054533, H20065986, H20066259, H20065443; National Food and Drug Administration National Drug Standard: WS-10001-(HD-0043)-2002, WS-10001-(HD-0043)-2002-2017, YBH18892006 | N |
| Xuebijing injection | Tianjin Chase Sun Pharmaceutical Co., Ltd. | Safflower, Red Paeoniae Trichocarpae, Szechwan Lovage Rhizome, Dan-shen Root, Chinese Angelica | *Carthamus tinctorius* L. [Asteraceae], *Paeonia lactiflora* Pall. [Paeoniaceae], *Conioselinum anthriscoides 'Chuanxiong'* [Apiaceae], *Salvia miltiorrhiza* Bunge [Lamiaceae],  *Angelica sinensis* (Oliv.) Diels [Apiaceae] | Warm-heat disease; systemic inflammatory response syndrome induced by infection; multiple organ dysfunction syndrome | Y-National Pharmaceutical Standard: Z20040033; National Food and Drug Administration National Drug Standard: YBZ01242004-2010Z-2012 | N |
| Chuanhuning injection | Beijing Sihuan Kebao Pharmaceutical Co., Ltd., Harbin Shengtai Bio-Pharmaceutical Co., Ltd., Chengdu Tongde Pharmaceutical Co., Ltd., Harbin Pharmaceutical Group Sanjing Jiabin Pharmaceutical Co., Ltd., Sinopharm Rongsheng Pharmaceutical Co., Ltd. | Andrographolide succinate half ester monopotassium salt | *Andrographis paniculata* (Burm.f.) Nees [Acanthaceae] | Viral pneumonia; viral upper respiratory tract infection | Y-National Pharmaceutical Standard: H20064574, H23022854, H51023408, H23023394, H20056897; National Food and Drug Administration National Drug Standard: YBH21282005 | N |
| Qingkailing injection | Yabao Pharmaceutical Group Co., Ltd., Guangzhou Baiyunshan Mingxing Pharmaceutical Co., Ltd., Shanxi Taihang Pharmaceutical Co., Ltd., Ji'an Yisheng Pharmaceutical Co., Ltd., Shenwei Pharmaceutical Group Co., Ltd. | Baicalin, cholic acid (animal drug), hyodeoxycholic acid (animal drug), Buffalo Horn (animal drug), Common Gardenia Fruit, Indigowoad Root, Japanese Honysuckle Flower Bud | *Scutellaria baicalensis* Georgi [Lamiaceae], Bos taurus domesticus Gmelin, Sus scrofa domestica Brisson, Bubali Cornu, *Gardenia jasminoides* J.Ellis [Rubiaceae], *Isatis tinctoria* L. [Brassicaceae], *Lonicera japonica* Thunb. [Caprifoliaceae] | Heat disease; acute hepatitis; upper respiratory tract infection; pneumonia; cerebral thrombosis; intracerebral hemorrhage | Y-National Pharmaceutical Standard: Z11020268, Z44022855, Z14021188, Z22026130, Z13020880; Execution standard: "Chinese Pharmacopoeia" 2015 Edition Part 1 | N |
| Shuanghuanglian injection | Harbin Zhenbao Pharmaceutical Co., Ltd.; Duoduo Pharmaceutical Co., Ltd.; Henan Fusen Pharmaceutical Co., Ltd.; Sichuan Shenwei Pharmaceutical Co., Ltd.; Heilongjiang Gerun Pharmaceutical Co., Ltd. | Japanese Honysuckle Flower Bud, Baikal Skullcap Root, Weeping Forsythia Fruit | *Lonicera japonica* Thunb. [Caprifoliaceae],  *Scutellaria baicalensis* Georgi [Lamiaceae],  *Forsythia suspensa* (Thunb.) Vahl [Oleaceae] | Fever, cough, and sore throat due to exogenous wind-heat; upper respiratory tract infection; pneumonia | Y-National Pharmaceutical Standard: Z23020785, Z23020795, Z41020750, Z20044485, Z20055309; Approval for Revision of National Drug Standards of the State Drug Administration: WS3-B-2104-96-2010; The Ministry of Health of the People's Republic of China, Chinese Medicine Prescriptions, Volume 11: WS3-B-2104-96 | N |
| Xiyanping injection | Jiangxi Qingfeng Pharmaceutical Co., Ltd. | Andrographolide sulfonate | *Andrographis paniculata* (Burm.f.) Nees [Acanthaceae] | Bronchitis; tonsillitis; bacillary dysentery | Y-National Pharmaceutical Standard: Z20026249; National Food and Drug Administration National Drug Standard: WS-10863(ZD-0863)-2002-2011-Z | N |
| Yuxingcao injection | Shanxi Jinxin Shuanghe Pharmaceutical Co., Ltd., Xi'an Qinba Pharmaceutical Co., Ltd., Jiangxi Poly Pharmaceutical Co., Ltd., Guangdong Xinfeng Pharmaceutical Co., Ltd., Chia Tai Qingchunbao Pharmaceutical Co., Ltd. | Heartleaf Houttuynia Herb | *Houttuynia cordata* Thunb. [Saururaceae] | Exuberance of heat-toxin; lung abscess; urinary tract infection | Y-National Pharmaceutical Standard: Z14021026, Z61020903, 236020399, Z44021272, Z33020013; National Food and Drug Administration National Drug Standard: WS3-B-3264-98 | N |

# File S6: Characteristics of the included studies.

| **Study ID** | **Sample Size (E/C)** | **Sex (M/F)** | **Age (Year, E/C)** | **Intervention in experimental group (WM+CHIs) ^*^** | **Intervention in** **control group (WM/WM+another CHIs) ^*^** | **Course of Treatment (Days)** | **Consistent baseline** | **Outcomes** | **Adverse drug reactions** |
| --- | --- | --- | --- | --- | --- | --- | --- | --- | --- |
| Bie XK 2018 | 28/28 | 29/27 | 5.01±1.32/4.62±1.25 | RDN 0.6 ml/kg+10%GS 100 ml, ivgtt, qd | Penicillin 100,000 U/kg, ivgtt; symptomatic treatment (ibuprofen) | 5 | Y | ①②③ | NR |
| Cai B 2013 | 39/39 | 56/22 | 25.5±12.6 | TRQ 0.5 ml/(kg·d)+5%GS 250 ml, ivgtt | Azithromycin; symptomatic supportive treatment (e.g., cooling) | 5 | Y | ①②⑥ | N |
| Cai YF 2011 | 60/58 | 62/56 | 1-14 | XBJ 10 ml+0.9%NS, ivgtt, qd | Antibiotic treatment (cephalosporins); symptomatic supportive treatment | 5 | Y | ①②⑤ | NR |
| Zeng GZ 2018 | 43/43 | 49/37 | 3.13±1.04/3.01±1.03 | XYP 0.1-0.2 ml/kg+0.9%NS/5%GS, ivgtt, qd | Symptomatic supportive treatment; anti-infective treatment | 5 | Y | ①② | NR |
| Zeng J 2012 | 92/92 | 110/74 | 2-14 | RDN 2-5(age): 0.5-0.8 ml/kg; 6-10: 10 ml; 11-14: 15 ml. +0.9%NS/5%GS 100-250 ml, ivgtt, qd | Mezlocillin sulbactam sodium 100mg/kg, ivgtt, bid; symptomatic treatment (ibuprofen) | 5 | Y | ① | NR |
| Che YL 2006 | 49/46 | 55/40 | 6/6 | TRQ 0.5-1 ml/kg+10%GS 100 ml, ivgtt, qd | Penicillin 200,000-300,000 U/(kg·d) ivgtt; symptomatic supportive treatment | 5-7 | Y | ① | NR |
| Chen WS 2014 | 16/16 | 19/13 | 10-48/12-49 | RDN+5%GS 150 ml, ivgtt, qd | Antibiotic treatment | - | Y | ① | NR |
| Chen X 2009 | 30/30 | 35/25 | 2-13 | RDN 1-5(age): 0.5-0.8 ml/kg; 6-10: 10 ml; 11-14: 15 ml. +0.9%NS/5%GS, ivgtt, qd | Cefuroxime 0.05 g/kg+0.9%NS 50-100 ml, ivggt, bid | 7 | Y | ①②④⑤⑥ | N |
| Chen YJ 2020 | 54/54 | 59/49 | 6.51±1.79/6.58±1.82 | RDN 1-5(age): 0.5-0.8 ml/kg; 6-10: 10 ml; 11-14: 15 ml. +0.9%NS 100-250 ml, ivgtt, qd | Cefuroxime 1.5 g+ 0.9%NS 100 ml, ivgtt, bid | 5 | Y | ①②④⑤ | NR |
| Deng WT 2015 | 55/55 | 66/44 | 29.3±6.1/30.9±5.9 | TRQ 20 ml+5%GS 250 ml, ivgtt, qd | Ceftezole sodium 4 g +0.9%NS 250 ml, ivggt, qd | 3-7 | Y | ① | NR |
| Ding JF 2010 | 50/50 | 65/35 | 2-14 | RDN 1-5(age): 0.5-0.8 ml/kg; 6-10: 10 ml; 11-14: 15 ml. +0.9%NS/5%GS, ivgtt, qd | Penicillin 100,000-200,000 U/kg+GS100-250 ml, ivgtt; cefodizime 25 mg/kg, ivggt, bid or fosfomycin 100 mg/kg ivggt, qd with positive penicillin skin test; symptomatic supportive treatment | 4 | Y | ①② | NR |
| Gao LL 2006 | 34/34 | 42/26 | 2-7 | YXC 1-2 ml/kg+5%GS 100-200 ml, ivgtt, qd | Penicillin 100,000 U/kg, ivgtt; symptomatic treatment (ibuprofen) | 3-5 | Y | ②⑤ | NR |
| Gao YS 2012 | 70/70 | 77/63 | ＜1: 4(number); 1-6: 35;＞6: 31/＜1: 13; 1-6: 34;＞6: 23 | YHN, ivgtt | Penicillin, ivgtt; symptomatic supportive treatment | 3-5 | Y | ① | NR |
| Ge LH 2015 | 53/53 | 55/51 | 2.6±1.1 | RDN 0.6 ml/kg+10%GS 100 ml, ivgtt, qd | Penicillin 100,000 U/kg, ivgtt; symptomatic treatment (ibuprofen) | 3-6 | Y | ①②③⑤ | NR |
| Ge MF 2014 | 54/54 | - | 8-20 | Penicillin, ivgtt, bid; RDN 8-10(age): 10 ml; 11-13: 15 ml; ＞13: 20 ml. +5%GS 250 ml, ivgtt, qd | Penicillin, ivgtt, bid; XYP 10mg/kg, ivgtt, qd | 3 | Y | ①⑥ | Detailed description |
| Ge WJ 2012 | 65/50 | 65/50 | 1-14 | YHN 8-10 mg/(kg·d), ivgtt, qd | Penicillin 100,000-200,000 U/(kg·d)+GS, ivgtt | 3-6 | Y | ②④⑤ | NR |
| Gong TY 2020 | 62/62 | 78/46 | 4.92±0.81/5.01±0.42 | XYP 2-4 ml+0.9%NS/5%GS, ivgtt | Symptomatic supportive treatment; anti-infective treatment | 3-7 | Y | ①②③④⑤ | NR |
| Gu JS 2019 | 45/45 | 45/45 | 4.5±1.2/4.5±1.2 | XYP 10mg/kg, ivgtt, qd | Clindamycin 5mg/kg, ivgtt, tid | 7 | Y | ①②③ | NR |
| Guo CP 2013 | 33/32 | 42/23 | 47.2±2.8/47.1±2.5 | TRQ 0.3-0.5 ml/kg+5%GS 100 ml, ivgtt | Penicillin 100,000 U/(kg·d)+5%GS 100ml, ivgtt; symptomatic supportive treatment | 7 | Y | ① | NR |
| Guo XY 2001 | 42/30 | 46/26 | 2-9 | YXC 1.5-2 ml/(kg·d), ivgtt, qd | Penicillin 100,000 U/(kg·d), ivgtt; symptomatic supportive treatment | 3-5 | Y | ①⑥ | Detailed description |
| He ML 2001 | 48/54 | 58/44 | 0.5-14 | CHN 10-15 mg/(kg·d)+10%GS, ivgtt, qd | Cefazolin sodium 60-100 mg/(kg·d)+10%GS, ivgtt, qd; symptomatic supportive treatment | 3 | Y | ① | NR |
| He Q 2015-1 | 60/60 | 81/39 | 5.5±1.2/5.8±1.3 | RDN 3-5(age): 0.5-0.8 ml/kg; 6-10: 10 ml; 11-14: 15 ml. +0.9%NS 50-100 ml, ivgtt, qd | Amoxicillin clavulanate potassium 30mg/kg+ 0.9%NS 50-100 ml, ivgtt, tid | 6 | Y | ①②⑤⑥ | N |
| He Q 2015-2 | 49/49 | 50/48 | 4.3±1.9/4.5±2.1 | TRQ+5%GS 250 ml, ivgtt, qd | Penicillin, ivgtt; symptomatic supportive treatment | - | Y | ①②⑤ | NR |
| He XL 2013 | 57/57 | 63/51 | 4.8±2.3/4.7±2.2 | XYP 5-10 mg/kg+5%GS 100 ml, ivgtt, qd | Clindamycin 5-8 mg/kg+0.9%NS 100 ml, ivgtt, tid; symptomatic supportive treatment | 7 | Y | ①⑥ | Detailed description |
| Hu QF 2017 | 100/100 | 112/88 | 5.65±2.07/5.54±1.85 | TRQ 0.3-0.5 ml/kg+0.9%NS, ivgtt, qd | Cefathiamidine 80mg/(kg·d), ivgtt, bid | 5 | Y | ①②③④⑤ | NR |
| Huang CQ 2014 | 48/48 | 49/47 | 18-45 | TRQ 20 ml+0.9%NS/5%GS 250 ml, ivgtt, qd | Cefathiamidine 2g+0.9%NS 100 ml, ivgtt, bid; symptomatic supportive treatment | 7 | Y | ①②③⑥ | N |
| Huang T 2018 | 35/35 | 42/28 | 32.9±3.9/33.6±4.1 | TRQ 20 ml+0.9%NS/5%GS 250 ml, ivgtt, qd | Cefathiamidine 2g+0.9%NS 100 ml, ivgtt, bid | 5 | Y | ①②③⑤ | NR |
| Jiang J 2012 | 38/38 | 42/34 | 18-65 | XYP 250 mg+0.9%NS 250 ml, ivgtt, qd | Cephalothin/penicillin; symptomatic supportive treatment | 5 | Y | ① | NR |
| Kuai HB 2013 | 26/26 | 28/24 | 6-42 | RDN 0.5-0.8 ml/kg+5%GS 150 ml, ivgtt, qd | Ceftizoxime sodium adult: 1-2g; children: 50 mg/kg. +5%GS, ivgtt, bid | 7 | Y | ①④⑤ | NR |
| Li AH 2019 | 35/35 | 43/27 | 5.64±1.32/5.72±1.38 | RDN 0.5-0.8 ml/kg+5%GS 100 ml, ivgtt, qd | Cefamandole sodium 50 mg/kg+0.9% NS 50 ml, ivgtt, bid; symptomatic supportive treatment | 3 | Y | ① | NR |
| Li F 2018 | 100/100 | 87/113 | ＜1: 6(number); 1-6: 50;＞6: 44/＜1: 19; 1-6: 49;＞6: 32 | YHN, ivgtt, qd | Cefuroxime sodium 50 mg/kg, ivgtt and then cefuroxime axetil 10 mg/kg, po; symptomatic supportive treatment | 3-5 | Y | ① | NR |
| Li HS 2020 | 50/50 | 49/51 | 25.33±3.14/25.16±3.27 | RDN 1 ml/kg+0.9%NS 100 ml, ivgtt, qd | Amoxicillin clavulanate potassium 1.2g, ivgtt, bid | 6 | Y | ①⑥ | Detailed description |
| Li HR 2020 | 40/40 | 48/32 | 22-59/15-62 | TRQ 20 ml+5%GS, ivgtt, qd | Penicillin, ivgtt, qd | 7 | Y | ①⑥ | Detailed description |
| Li JJ 2017 | 54/54 | 58/50 | 6.3±1.5/5.9±1.3 | XYP 5-10 mg/kg, ivgtt, qd | Cefuroxime sodium 100 mg/kg, ivgtt, bid; symptomatic supportive treatment | 7 | Y | ①②⑤ | NR |
| Li J 2004 | 60/66 | 69/57 | ＜1: 8(number); 1-6: 28; ＞6: 24/＜1: 12; 1-6: 30; ＞6: 24 | XYP 0.2-0.4 ml/(kg·d)+5%GS, ivgtt | Penicillin 200,000-300,000 U/(kg·d), ivgtt, bid | 5-7 | Y | ① | NR |
| Li Q 2013 | 18/18 | 21/15 | 26.5±4.8 | RDN 0.5-0.8 ml/kg+15%GS 150 ml, ivgtt, qd | Antibiotic treatment | 7 | Y | ① | NR |
| Li X 2015 | 100/100 | 126/74 | 25.3/26.2 | RDN, ivgtt, qd | Anti-infective treatment; symptomatic supportive treatment | 5 | Y | ① | NR |
| Li ZX 2012 | 30/30 | 39/21 | 4.22±1.75/4.22±1.88 | XBJ 1 ml/ (kg·d)+0.9%NS 100-250 ml, ivgtt, qd | Antibiotic treatment | 5 | Y | ① | NR |
| Lin S 2002 | 40/36 | 41/35 | 24/22 | QKL 20-40 ml+5%-10%GS/0.9%NS, ivgtt | Cephazolin sodium/ penicillin/lincomycin, ivgtt | 3 | Y | ①⑥ | Detailed description |
| Liu H 2014 | 28/28 | 29/27 | 3-51 | RDN+5%GS/0.9%NS 150ml, ivgtt, adult: bid; children: qd | Penicillin, ivgtt, bid | - | Y | ① | NR |
| Liu PH 2008 | 36/33 | 38/31 | 1-14 | XYP 5-10 mg/(kg·d)+5%GS 100-250 ml, ivgtt, qd | Penicillin 200,000 U/(kg·d), ivgtt, bid | 3-5 | Y | ①⑥ | N |
| Liu YH 2020 | 43/43 | 42/44 | 6.01±0.77/5.67±0.82 | XYP 5 mg/(kg·d)+5%GS 100 ml, ivgtt, qd | Amoxicillin clavulanate potassium 30mg/kg+0.9%NS 100 ml, ivgtt, tid | 5 | Y | ⑥ | Detailed description |
| Long DL 2015 | 35/35 | 42/28 | 6.12±2.28/5.88±2.45 | SHL 60 mg/kg+5%GS 500 ml, ivgtt, qd | Penicillin 250,000 U/kg, ivgtt, q12h | 7 | Y | ①②③⑤⑥ | N |
| Long QB 2014 | 30/30 | 35/25 | 4.86±0.27 | XYP 0.2 ml/kg+GS 50-100 ml, ivgtt, qd | Mezlocillin sulbactam 75 mg/kg+5%GS 100 ml, ivgtt, bid; symptomatic supportive treatment | - | Y | ①⑥ | Detailed description |
| Lu SQ 2010 | 60/60 | 65/55 | 2-11, 6.1(mean) | XYP 5-10 mg/(kg·d)+5%GS 250 ml, ivgtt, qd | Amoxicillin clavulanate potassium 30mg/kg+0.9%NS 100 ml, ivgtt, q8h | 7 | Y | ①⑥ | N |
| Luo LY 2003 | 30/30 | 42/18 | 2-7 | YXC 1-2ml/(kg·d), ivgtt, qd | Cefradine 50-100 mg/(kg·d), ivgtt, bid or oxacillin 50-100 mg/(kg·d), ivgtt, bid; symptomatic supportive treatment | 3 | Y | ①⑥ | N |
| Luo W 2016 | 49/49 | 59/39 | 4.5±2.1/4.6±2.1 | XYP 5-10 mg/kg +5%GS 100 ml, ivgtt, qd | Clindamycin 5-8 mg/kg+0.9%NS 100 ml, ivgtt, tid | 7 | Y | ① | NR |
| Lv Y 2020 | 60/60 | 68/52 | 6.04±3.28 | XYP 5-10 mg/kg +5%GS 250 ml, ivgtt, qd | Beta-lactam antibiotics, ivgtt; symptomatic supportive treatment | 5 | Y | ①③④ | NR |
| Ma JM 2015 | 48/48 | 51/45 | 6.8±1.7/6.3±1.4 | XYP 5-10 mg/kg +5%GS 250 ml, ivgtt, qd | Azithromycin 10 mg/kg+5%GS 500 ml, ivgtt, qd | 5 | Y | ① | NR |
| Ma N 2013 | 80/70 | 98/52 | 1-12/1-12 | RDN 0.6 ml/(kg·d)+5%GS 100-150ml, ivgtt, qd | Penicillin 200,000 U/(kg·d)+0.9%NS 100-150 ml, ivgtt, bid | 5-10 | Y | ①②⑤⑥ | N |
| Niu ZY 2005 | 38/32 | - | 2.5-8, 5.6(mean)/2-7.5, 5.2 | TRQ 0.3-0.5 ml/kg+10%GS 100-250 ml, ivgtt, qd | Penicillin 200,000 U/kg+0.9%NS 100 ml, ivgtt, qd | 5 | Y | ① | NR |
| Ouyang XR2017 | 100/100 | 104/96 | 4.77±2.13/4.71±2.38 | XYP 0.2-0.4 ml/(kg·d)+5%GS, ivgtt | Beta-lactam antibiotics, ivgtt; symptomatic supportive treatment | 5 | Y | ①②③④⑤⑥ | N |
| Pan LL 2014-1 | 60/60 | 63/57 | 2-13 | XYP 5-10 mg/(kg·d) +5%GS 250 ml, ivgtt, qd | Azithromycin 10 mg/(kg·d)+5%GS 500 ml, ivgtt, qd | 5 | Y | ① | NR |
| Pan LL 2014-1 | 60/60 | 66/54 | 4.21±1.88/4.22±1.83 | Azithromycin 10 mg/(kg·d)+5%GS 500 ml, ivgtt, qd; XYP 5-10 mg/(kg·d)+5%GS 250 ml, ivgtt, qd | Azithromycin 10 mg/(kg·d)+5%GS 500 ml, ivgtt, qd; TRQ 0.3-0.5 ml/(kg·d)+5%GS 250 ml, ivgtt, qd | 5 | Y | ① | NR |
| Peng XD 2015 | 50/50 | 55/45 | 5.2±2.1/5.5±2.3 | XYP 5-10 mg/kg+5%GS 250 ml, ivgtt, qd | Clindamycin 25-40 mg/kg+0.9%NS 500 ml, ivgtt, qd | 3 | Y | ①⑥ | N |
| Qiao Y 2015 | 65/61 | 71/55 | 3.96±2.37/4.05±2.51 | XYP 5 mg/(kg·d) +5%GS 100 ml, ivgtt, qd | Beta-lactam antibiotics, ivgtt; symptomatic supportive treatment | 7-10 | Y | ①②③④⑤⑥ | Detailed description |
| Qiu L 2019 | 63/63 | 76/50 | 2.30±1.20/2.35±1.30 | RDN 0.6 ml/kg+10%GS 100ml, ivgtt, qd | Penicillin 100,000 U/kg, ivgtt; symptomatic supportive treatment | 3 | Y | ①②③④⑤⑥ | Detailed description |
| Ren LL 2016 | 45/45 | 50/40 | 6±3.2/6±3.8 | RDN 1-5(age): 0.5-0.8 ml/kg; 6-10: 10 ml; 11-14: 15 ml. +5%GS 100-250 ml, ivgtt, qd | Cefuroxime sodium 100 mg/kg+0.9%NS 50-100 ml, ivgtt, bid | 5 | Y | ①②⑤⑥ | N |
| Ren XJ 2018 | 47/47 | 49/45 | 5.32±2.21/5.28±2.14 | XYP 250mg+5%GS, ivgtt, qd | Clindamycin 15 mg/(kg·d), ivgtt, tid | 3 | Y | ①⑥ | Detailed description |
| Shen Y 2015 | 100/100 | 100/100 | 7.2 ±1.5 | RDN 2-5(age): 0.5-0.8 ml/kg; 6-10: 10 ml; 11-15: 15 ml. +0.9%NS/5%GS 100-250 ml, ivgtt, qd | Mezlocillin sulbactam 100 mg/kg, ivgtt, bid; symptomatic supportive treatment (ibuprofen) | 5 | Y | ① | NR |
| Shi LC 2018 | 450/450 | 495/405 | 4.88±0.34/4.98±0.35 | XYP 0.2-0.4 ml/(kg·d)+GS 50ml, ivgtt | Beta-lactam antibiotics, ivgtt; symptomatic supportive treatment | 5 | Y | ①②③④⑤ | NR |
| Shi YP 2006 | 28/20 | - | 8-50 | TRQ adult: 20 ml/d, children: 0.5 ml/(kg·d), ivgtt | Cefotaxime sodium 50-100 mg/(kg·d)+0.9%NS/5%GS 250 ml, ivgtt | 3-5 | Y | ① | NR |
| Shuai CR 2018 | 54/54 | 67/41 | 4.2±1.0/4.7±1.4 | XYP 10 mg/kg, ivgtt, qd | Clindamycin 5 mg/kg, ivgtt, tid | 7 | Y | ①②③⑥ | Detailed description |
| Song HH 2020 | 37/37 | 41/33 | 24.03±5.27/24.18±5.11 | RDN 20 ml+0.9%NS 250 ml, ivgtt, qd | Penicillin 4,000,000 U+0.9%NS 100 ml, ivgtt, bid, or  lincomycin 0.6 g+0.9%NS 100 ml, ivgtt, bid with positive penicillin skin test; symptomatic supportive treatment | 4 | Y | ①⑥ | Detailed description |
| Song YY 2018 | 68/75 | 73/70 | 8.14±0.23 | XYP 0.2 ml/(kg·d), ivgtt, qd | Cefuroxime 80 mg/(kg·d)+0.9%NS 100 ml, ivgtt, bid; symptomatic supportive treatment | - | Y | ②⑤⑥ | Detailed description |
| Su P 2015 | 30/30 | 26/34 | 6.7±2.3/6.8±2.1 | YHN, ivgtt, qd | Penicillin100,000-200,000 U/(kg·d)+GS, ivgtt, bid, or amoxicillin clavulanate potassium 50-70mg/(kg·d)+GS, ivgtt, bid; symptomatic supportive treatment | 4 | Y | ①② | NR |
| Sun T 2020 | 254/105 | 258/101 | 11.06±2.57/11.15±2.62 | XYP 5-10 mg/kg+5%GS 250 ml, ivgtt, qd | Cefprozil 7.5 mg/(kg·d), po, bid or 8 mg/(kg·d), po, qd | 7 | Y | ①②③⑤⑥ | Detailed description |
| Tang Y 2008 | 50/50 | 62/38 | 20-44/21-43 | TRQ 20 ml+5%GS 250 ml, ivgtt, qd | Penicillin, ivgtt; symptomatic supportive treatment | 4 | Y | ①②③⑥ | N |
| Wang HT 2015 | 39/39 | 45/33 | 5.19±2.34/5.25±2.17 | XYP 5-10 mg/kg+5%GS 100 ml, ivgtt, qd | Clindamycin 5-8 mg/kg+0.9%NS 100 ml, ivgtt, tid; symptomatic supportive treatment | 7 | Y | ①⑥ | N |
| Wang JJ 2016 | 57/46 | 52/51 | 2-38/4-42 | XYP 5 mg/kg+0.9%NS/5%GS, ivgtt, qd | Mezlocillin sodium 75 mg/kg+0.9%NS 250 ml, ivgtt, bid; symptomatic supportive treatment | 3 | Y | ①②③④ | NR |
| Wang W 2018 | 25/25 | 33/17 | 41.8±3.2/41.2±3.1 | XYP 250 mg +0.9%NS 250ml, ivgtt, qd | Beta-lactam antibiotics, ivgtt; symptomatic supportive treatment | 5 | Y | ① | NR |
| Wang Y 2014 | 125/125 | 152/98 | 7.1±0.9 | RDN 2-5(age): 0.5-0.8 ml/kg; 6-10: 10 ml; 11-14: 15 ml. +0.9%NS/5%GS 100-250 ml, ivgtt, qd | Mezlocillin sulbactam 100 mg/kg, ivgtt, bid; symptomatic supportive treatment | 5 | Y | ① | NR |
| Wang Y 2015 | 64/64 | 82/46 | 6.38±4.12/6.85±4.38 | RDN ＜3(age): 0.5 ml/(kg·d);3-5: 0.8 ml/(kg·d); ＞5: 10 ml. +0.9%NS 100 ml, ivgtt, qd | Azithromycin 10 mg/(kg·d)+5%GS 500 ml, ivgtt, qd | 5 | Y | ① | NR |
| Wang YY 2020 | 33/32 | 37/28 | 2.80±1.43/2.90±1.42 | XYP 2-4 ml+0.9%NS/5%GS, ivgtt, qd | Anti-infective treatment; symptomatic supportive treatment | 3-7 | Y | ①②③④⑤ | NR |
| Wei RY 1998 | 50/40 | 60/30 | ≤3: 10(number); ＞3: 40/≤3: 14; ＞3: 26 | QKL≤3(age): 8-10 ml; 3-7:10-20 ml; ＞7: 20-30 ml, ivgtt, qd | Penicillin100,000-200,000 U/(kg·d), ivgtt | 7 | Y | ① | NR |
| Wu B 2017 | 48/48 | 51/45 | 4.3±1.5/4.4±1.7 | RDN 3-5(age): 0.6 ml/kg; 6-10: 10 ml; 11-12: 15 ml. +5%GS 100-250 ml, ivgtt, qd | Cefathiamidine, ivgtt; symptomatic supportive treatment | 5 | Y | ①②④⑤⑥ | Detailed description |
| Xie BX 2013 | 43/43 | 45/41 | 22-48/22-59 | TRQ 0.3-0.5 ml/kg+0.9%NS/5%GS 250 ml, ivgtt, qd | Penicillin, ivgtt; symptomatic supportive treatment | 5 | Y | ① | NR |
| Xin L 2021 | 30/30 | 32/28 | 31.51±10.83/35.67±10.34 | XYP 10 ml+5%GS 100 ml, ivgtt, qd | Cefoxitin sodium 4g+0.9%NS 250 ml, ivgtt, qd | 2-5 | Y | ①②③⑥ | N |
| Xu J 2017 | 50/50 | 55/45 | 3-13 | RDN 0.5 ml/kg+5%GS 100-250 ml, ivgtt, qd | Beta-lactam antibiotics, ivgtt; symptomatic supportive treatment | 5-7 | Y | ⑥ | N |
| Yang BX 2015 | 50/50 | 65/35 | 4.12±1.54/4.24±1.43 | XYP 5-10 mg/(kg·d)+5%GS 250ml, ivgtt, qd | Azithromycin 10 mg/(kg·d)+5%GS 500 ml, ivgtt, qd | 5 | Y | ①⑥ | Detailed description |
| Wu XZ 2013 | 60/60 | 72/48 | 4.8 ±1.5/4.3 ±1.8 | XYP 0.2-0.4 ml/(kg·d), ivgtt, qd | Mezlocillin sulbactam 75 mg/kg, ivgtt, bid; symptomatic supportive treatment | 5 | Y | ① | NR |
| Yang B 2010 | 38/40 | 40/38 | 2-12/2-12 | TRQ 0.3-0.5 ml/kg+5%GS 30-50 ml, ivgtt, qd | Penicillin 200,000 U/(kg·d), ivgtt, bid-tid; symptomatic supportive treatment | 3-5 | Y | ①②⑤ | NR |
| Yang L 2012 | 32/32 | 35/29 | 36.63±11.19/34.94±13.40 | RDN 20 ml+0.9%NS 250 ml, ivgtt, qd | Penicillin 4,000,000 U/(kg·d)+0.9%NS 100 ml, ivgtt, bid or cefoxitin sodium 1.5g+0.9%NS 20 ml, ivgtt, bid, or lincomycin 0.6g+0.9%NS 100-200 ml, ivgtt, q8h/q12h with positive penicillin skin test; symptomatic supportive treatment | 4 | Y | ① | NR |
| Yang WH 2009 | 30/35 | 46/19 | 4.22±1.89/4.22±1.76 | TRQ 0.3-0.5 ml/(kg·d)+5%GS 250 ml, ivgtt, qd | Cefotaxime sodium 100mg/(kg·d)+0.9%NS 250 ml, ivgtt | 5 | Y | ① | NR |
| Yang Y 2008 | 62/60 | 70/52 | 2-6: 42(number); 7-14: 16; ＞14: 4/2-6: 40; 7-14: 14; ＞14: 6 | YHN 10 mg/(kg·d)+GS, ivgtt, qd | Penicillin 100,000-200,000 U/(kg·d)+GS, ivgtt, bid or Amoxicillin clavulanate potassium50-70 mg/(kg·d)+GS, ivgtt, bid; symptomatic supportive treatment | 3-5 | Y | ① | NR |
| Yu CM 2016 | 52/52 | 53/51 | 3-6: 31(number); 7-12: 21/3-6: 32; 7-12: 20 | XBJ 20 ml+0.9%NS 100 ml, ivgtt, bid | Clindamycin 25-40 mg/kg+5%GS, ivgtt, bid | 5 | Y | ①②③④⑤⑥ | N |
| Yu LL 2015 | 34/34 | 38/30 | 7.5±4.2/7.5±3.6 | TRQ 20 ml+5%GS 500 ml, ivgtt, qd | Azithromycin 10 mg/(kg·d), ivgtt, qd | 3 | Y | ①②⑤ | NR |
| Yu ZH 2011 | 42/40 | 44/38 | 23±16.15/24±13.25 | TRQ 20 ml+5%GS 250 ml, ivgtt, qd | Ceftezole sodium 4g+0.9%NS 250 ml, ivgtt, qd; symptomatic supportive treatment | 3-7 | Y | ① | NR |
| Yuan F 2018 | 41/41 | 51/31 | 5.68±1.23/5.56±1.12 | RDN ＜5(age): 0.6 ml/kg; 6-10: 10 ml; ＞11: 15 ml. +GS 100-250 ml, ivgtt, qd | Cefathiamidine 2 g+0.9%NS 100 ml, ivgtt, bid; symptomatic supportive treatment | 5 | Y | ①⑥ | Detailed description |
| Yuan XY 2000 | 30/30 | 25/35 | 4-10 | SHL 60 mg/kg+10%GS 100-300 ml, ivgtt, qd | Penicillin 100,000 U/kg, ivgtt; symptomatic supportive treatment | 3-5 | Y | ②⑤⑥ | Detailed description |
| Zhang GP 2013 | 60/40 | 60/40 | 56.5±1.5 | TRQ 20 ml+0.9%NS 250 ml, ivgtt, qd | Azithromycin 0.5 g+5%GS 250 ml, ivgtt, qd | 7 | Y | ① | NR |
| Zhang HQ 2013 | 76/74 | 80/70 | 6-14/6-14 | YHN 8-10 mg/kg+GS, ivgtt | Amoxicillin clavulanate potassium, or azithromycin/roxithromycin with positive penicillin skin test | 5 | Y | ① | NR |
| Zhang LY 2005 | 40/40 | 42/38 | 2-12, 7(mean) | TRQ 0.3-0.5 ml/(kg·d)+5%GS 250 ml, ivgtt, qd | Penicillin, ivgtt | 5 | Y | ①②⑥ | N |
| Zhang L 2007 | 53/50 | 67/36 | 1-7 | RDN 0.5-0.8 ml/kg+10%GS 100 ml, ivgtt, qd | Anti-infective treatment; symptomatic supportive treatment | 3 | Y | ①⑥ | N |
| Zhang WC 2016 | 50/50 | 61/39 | 6.7±0.8/7.0±0.6 | RDN 1-5(age): 0.5-0.8 ml/kg; 6-10: 10 ml+5%GS 50-100 ml, ivgtt, qd | Amoxicillin clavulanate potassium 30 mg/kg+0.9%NS 100 ml, ivgtt, q12h | 7 | Y | ①②③④⑤⑥ | Detailed description |
| Zhang X 2017 | 43/43 | 49/37 | 4.3±1.0/4.1±1.2 | XYP 5-10 mg/kg+5%GS 500ml, ivgtt, qd | Azithromycin 10 mg/kg+5%GS 500 ml, ivgtt, qd; symptomatic supportive treatment | 5 | Y | ①②③ | NR |
| Zhang ZW 2021 | 60/60 | 66/54 | 4.51±0.82/3.94±0.97 | TRQ 0.3-0.5 ml/kg+5%GS 100-200 ml, ivgtt, qd | Cefuroxime sodium 100 mg/(kg·d)+0.9%NS 50ml, ivgtt, bid; adenine arabinoside 5-10 mg/(kg·d)+5%GS 100 ml, ivgtt, qd; symptomatic supportive treatment | 7 | Y | ①②③⑥ | Detailed description |
| Zhao CQ 2014 | 50/50 | 53/47 | 7.7±3.0/7.3±3.1 | RDN 2-5(age): 0.5-0.8 ml/kg; 6-10: 10 ml; 10-13: 15 ml+0.9%NS 100-200 ml, ivgtt, qd | Azithromycin 10 mg/kg+0.9%NS 100 ml, ivgtt, qd | - | Y | ① | NR |
| Zhao MD 2014 | 35/35 | 39/31 | 1-12, 5.3(mean)/9-13, 5.4 | SHL 60 mg/(kg·d)+5%GS 500 ml, ivgtt, qd | Penicillin 5,000-20,000 U/(kg·d), ivgtt, bid; symptomatic supportive treatment | 5-7 | Y | ①②③⑤ | NR |
| Zhao QH 2018 | 36/38 | 39/35 | 4.4±1.9/4.6±2.2 | TRQ 50 ml+5%GS 250 ml, ivgtt, qd | Penicillin 200,000 U/(kg·d), ivgtt, tid; symptomatic supportive treatment | 3 | Y | ①②③ | NR |
| Zhao XS 2015 | 34/33 | 41/26 | 5.2±0.9 | SHL 60 mg/(kg·d)+5%GS 500 ml, ivgtt, qd | Penicillin 5,000-20,000 U/(kg·d), ivgtt, bid; symptomatic supportive treatment | 5-7 | Y | ①②③ | NR |
| Zhao Y 2006 | 30/30 | 25/35 | 4-10 | QKL 0.5-0.8 ml/kg+10%GS 100 ml, ivgtt, qd | Penicillin 100,000 U/kg, ivgtt; symptomatic supportive treatment | 3-5 | Y | ②⑤⑥ | Detailed description |
| Zhou HF 2003 | 100/88 | 101/87 | 1-3:61(number); 4-8:96; 9-12:31 | YXC 1-1.5ml/(kg·d)+5%GS, ivgtt, qd | Penicillin, ivgtt; symptomatic supportive treatment | 5 | Y | ① | NR |
| Zhou KL 2018 | 47/43 | 52/38 | 5.46±2.39/5.18±2.65 | XYP 5-10 mg/kg+5%GS 250ml, ivgtt, qd | Penicillin 1,000,000-3,000,000 U, ivgtt, bid; symptomatic supportive treatment | 5 | Y | ① | NR |
| Zhou SL 2013 | 112/118 | 119/111 | 4.9±1.2/4.7±1.3 | RDN 0.6 ml/kg+10%GS 100 ml, ivgtt, qd | Penicillin 100,000 U/kg, ivgtt; symptomatic supportive treatment | 3 | Y | ①②③④⑤⑥ | Detailed description |
| Zhou XP 2019 | 60/60 | 66/54 | 5.3±2.2/5.4±2.1 | XYP 5-10 mg/kg+5%GS 250ml, ivgtt, qd | Clindamycin 25-40 mg/kg+0.9%NS 500 ml, ivgtt, qd | 7 | Y | ①②③④⑤ | NR |
| Zhu XX 2014 | 89/83 | 94/78 | 2-5/2-5 | RDN 0.6-0.8 ml/(kg·d)+5%GS, ivgtt, qd | Ceftriaxone 80mg/kg, ivgtt; symptomatic supportive treatment | 6 | Y | ① | NR |
| Zhu HL 2017 | 39/39 | 47/31 | 26.18±2.13/26.67±2.01 | TRQ 0.3-0.5 ml/kg+0.9%NS, ivgtt, qd | Cefathiamidine 80mg/(kg·d), ivgtt, bid | 5 | Y | ① | NR |
| Zou AG 2010 | 30/30 | 37/23 | 1-6: 18(number); ＞6: 12/1-6: 19; ＞6: 11 | XYP 0.2-0.4ml/(kg·d)+5%GS, ivgtt, qd | Penicillin 200,000 U/(kg·d), ivgtt, bid; symptomatic supportive treatment | 3-5 | Y | ①⑥ | N |
| Zou R 2011 | 65/66 | 65/66 | 1-6: 45(number); ＞6: 20/1-6: 43; ＞6: 23 | YHN 5-10g/(kg·d)+0.9%NS 100-250 ml, ivgtt, qd | Penicillin 200,000 U/(kg·d), ivgtt, qd; mezlocillin sulbactam 125 mg/(kg·d), ivgtt, qd; symptomatic supportive treatment | 3-5 | Y | ①⑥ | Detailed description |

Note: ^*^ The groups received the same treatment regimens of WM; E/C, experimental group/control group; M/F, male/female; CHIs, Chinese herbal injections; WM, Western Medicine; NR, Not Reported; N, No; RDN, Reduning injection; TRQ, Tanreqing injection; QKL, Qingkailing injection; XBJ, Xuebijing injection; SHL, Shuanghuanglian injection; YHN, Yanhuning injection; CHN, Chuanhuning injection; YXC, Yuxingcao injection; XYP, Xiyanping injection; ①, Clinical effectiveness rate; ②, Antipyretic time; ③, Sore throat relief time; ④, Red and swollen tonsils relief time; ⑤, Tonsillar exudate relief time; ⑥, Adverse drug reactions.

# File S7: Forest plot of inconsistency in clinical effectiveness rate.


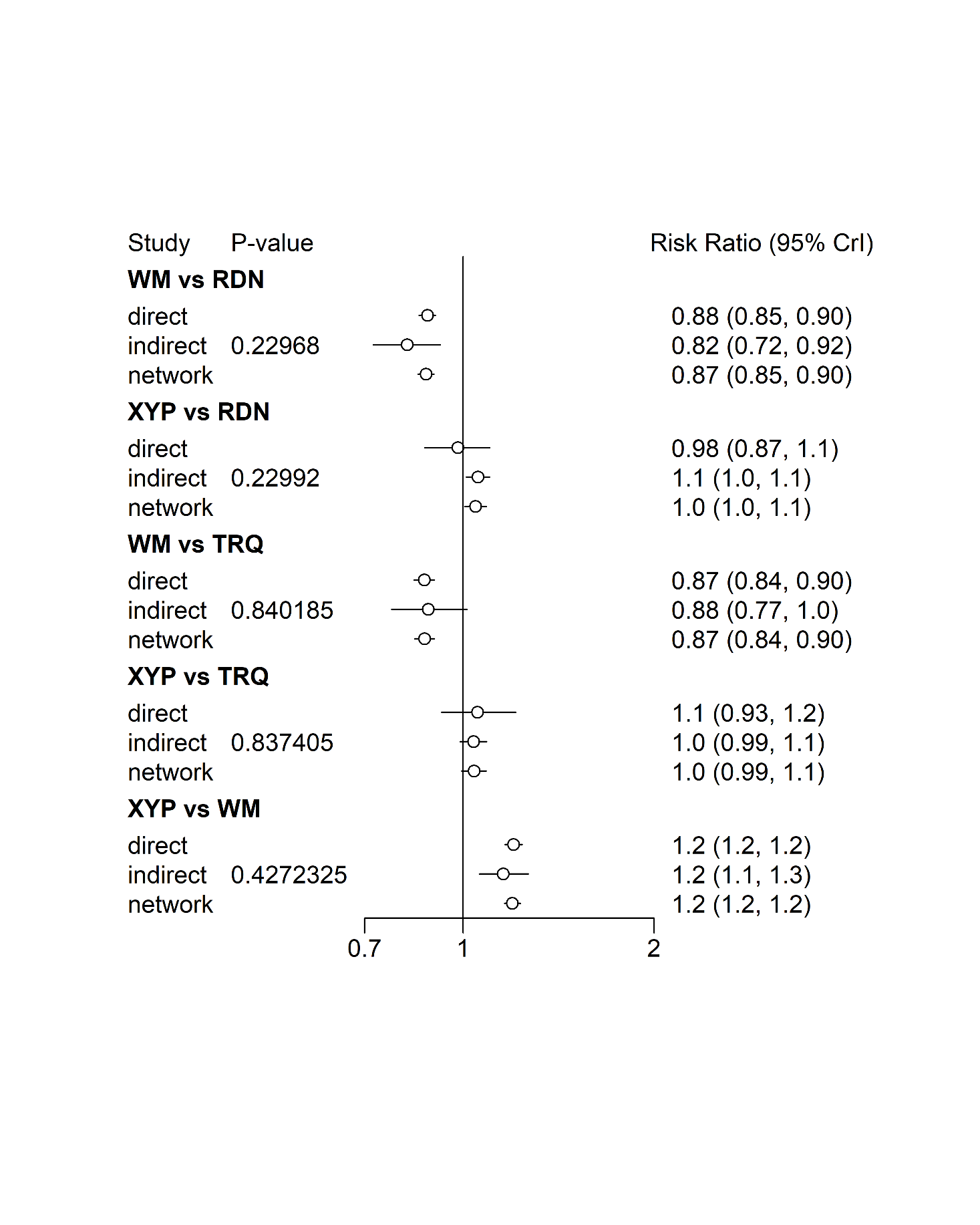


Note: WM, Western Medicine; RDN, Reduning injection; TRQ, Tanreqing injection; XYP, Xiyanping injection.

# File S8: Heatmap for contribution degree of inconsistency in clinical effectiveness rate.


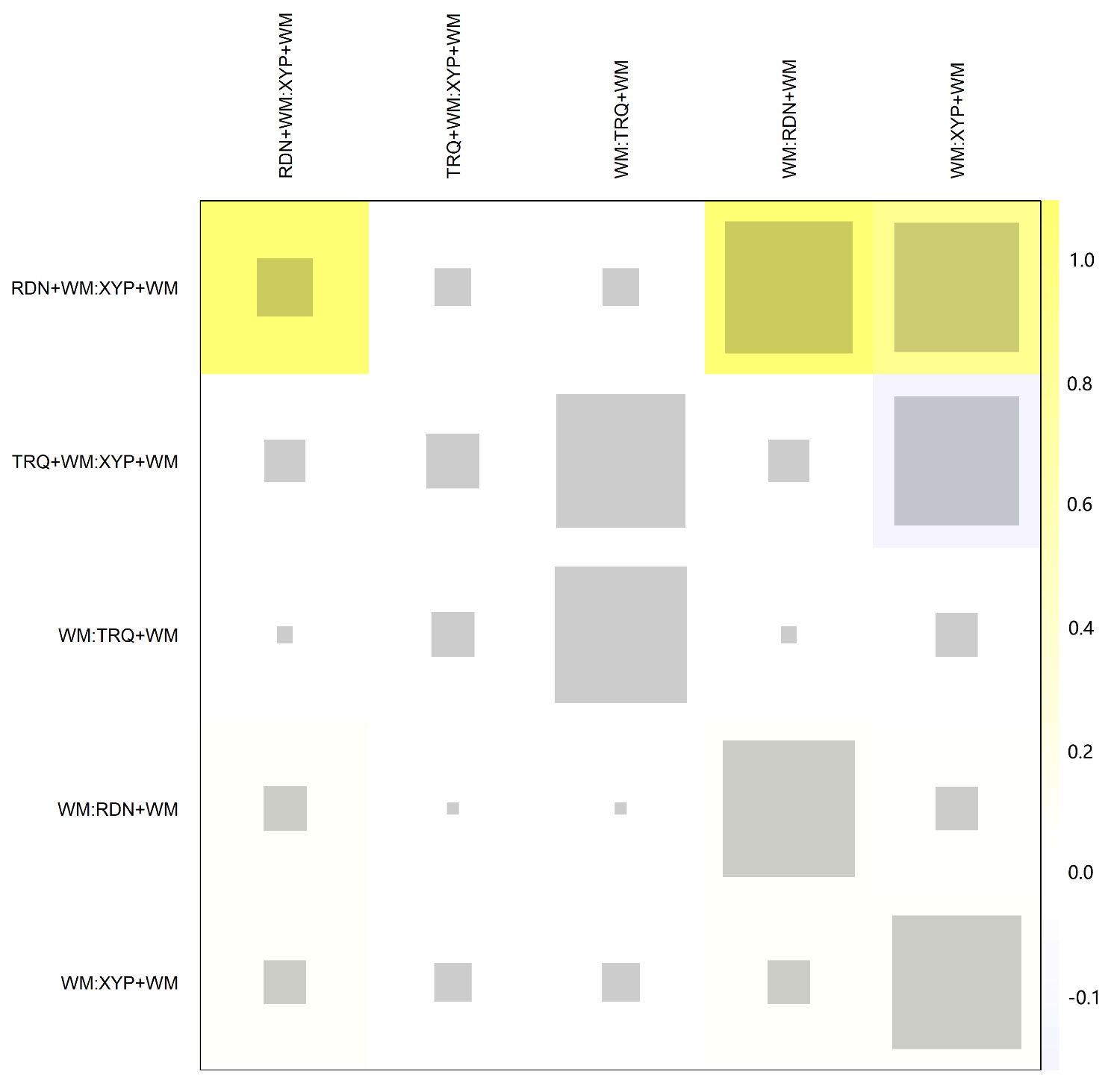


Note: The yellower the color, the greater the contribution to the inconsistency; results within each line represent the pooled comparisons; WM, Western Medicine; RDN, Reduning injection; TRQ, Tanreqing injection; XYP, Xiyanping injection.

# File S9: Results of Egger’s test.

## Egger’s test for clinical effectiveness rate.

| **Std_Eff** | **Coef.** | **Std. Err.** | **t** | **P>\|t\|** | **[95% Conf. Interval]** | | **P-value** |
| --- | --- | --- | --- | --- | --- | --- | --- |
| **slope** | .0298588 | .036277 | 0.82 | 0.412 | -.042105 | .1018227 | 0.434 |
| **bias** | -.463519 | .5902825 | -0.79 | 0.434 | -1.634481 | .7074426 |  |

## Egger’s test for antipyretic time.

| **Std_Eff** | **Coef.** | **Std. Err.** | **t** | **P>\|t\|** | **[95% Conf. Interval]** | | **P-value** |
| --- | --- | --- | --- | --- | --- | --- | --- |
| **slope** | -1.253154 | .380358 | -3.29 | 0.002 | -2.018775 | -.4875331 | 0.006 |
| **bias** | 8.451205 | 8.451205 | 2.91 | 0.006 | 2.602041 | 14.30037 |  |

## Egger’s test for sore throat relief time.

| **Std_Eff** | **Coef.** | **Std. Err.** | **t** | **P>\|t\|** | **[95% Conf. Interval]** | | **P-value** |
| --- | --- | --- | --- | --- | --- | --- | --- |
| **slope** | -.5395078 | .4808863 | -1.12 | 0.272 | -1.527984 | .4489681 | 0.360 |
| **bias** | 4.012333 | 4.306422 | 0.93 | 0.360 | -4.839645 | 12.86431 |  |

## Egger’s test for red and swollen tonsils relief time.

| **Std_Eff** | **Coef.** | **Std. Err.** | **t** | **P>\|t\|** | **[95% Conf. Interval]** | | **P-value** |
| --- | --- | --- | --- | --- | --- | --- | --- |
| **slope** | -.2893951 | .3775207 | -0.77 | 0.455 | -1.089703 | .5109131 | 0.424 |
| **bias** | 2.783023 | 3.390151 | 0.82 | 0.424 | -4.403777 | 9.969823 |  |

## Egger’s test for tonsillar exudate relief time.

| **Std_Eff** | **Coef.** | **Std. Err.** | **t** | **P>\|t\|** | **[95% Conf. Interval]** | | **P-value** |
| --- | --- | --- | --- | --- | --- | --- | --- |
| **slope** | -.2678388 | .4552703 | -0.59 | 0.561 | -1.196369 | .660691 | 0.400 |
| **bias** | 2.847419 | 3.338269 | 0.85 | 0.400 | -3.961026 | 9.655864 |  |

# File S10: Forest plots of network meta-regression.


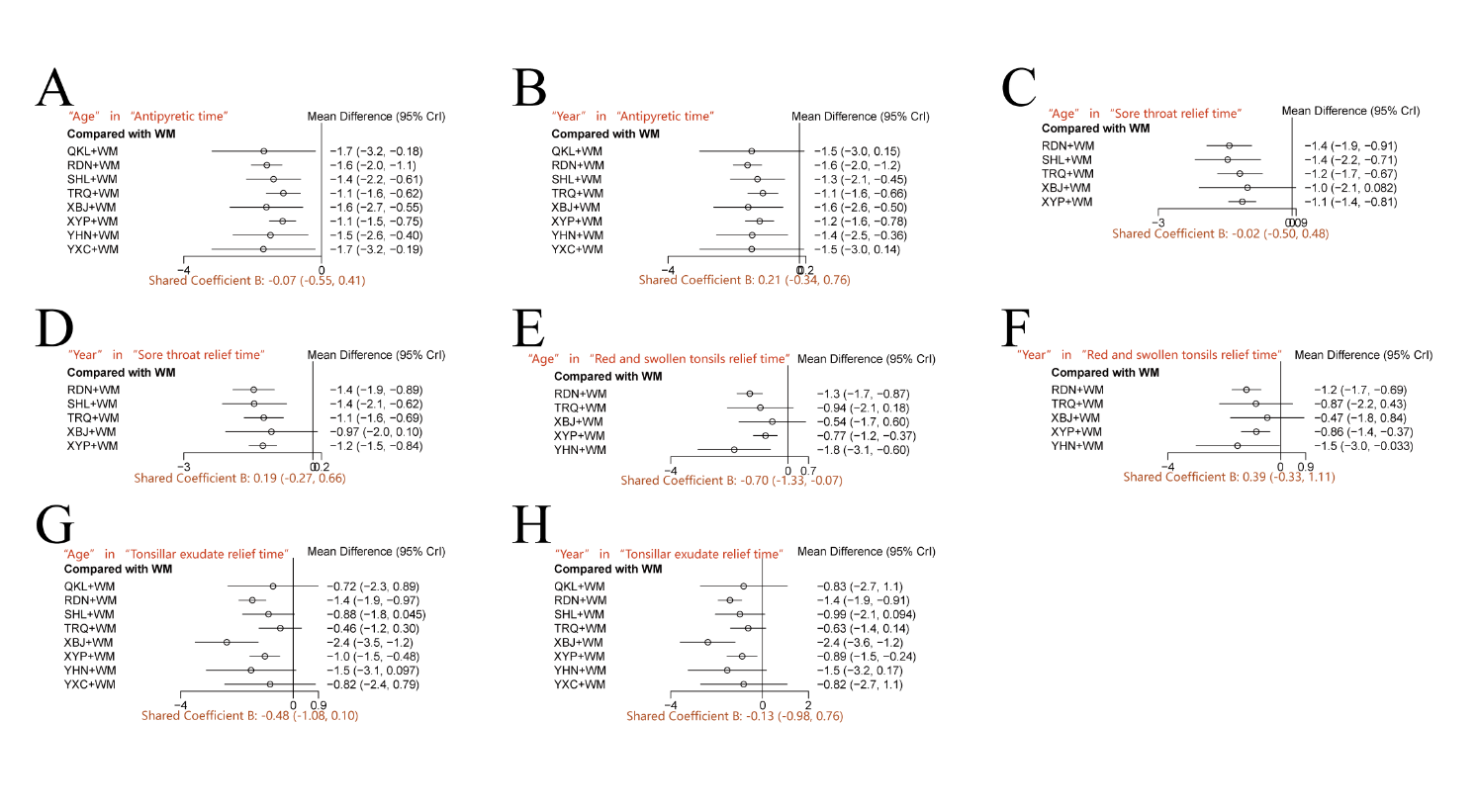


Note: WM, Western Medicine; RDN, Reduning injection; TRQ, Tanreqing injection; QKL, Qingkailing injection; XBJ, Xuebijing injection; SHL, Shuanghuanglian injection; YHN, Yanhuning injection; YXC, Yuxingcao injection; XYP, Xiyanping injection.

# File S11: Sensitivity analysis.

|  | **Clinical effectiveness rate, RR (95% CI)** | **Antipyretic time, MD (95% CI)** | **Sore throat relief time, MD (95% CI)** | **Red and swollen tonsils relief time, MD (95% CI)** | **Tonsillar exudate relief time, MD (95% CI)** |
| --- | --- | --- | --- | --- | --- |
| **Ranking probabilities of SUCRA** | XBJ+WM ranked first (81.23%)/QKL+WM was not included in this outcome analysis | RDN+WM ranked first (71.33%) | SHL+WM ranked first (67.88%) | YHN+WM ranked first (89.41%) | XBJ+WM ranked first (91.46%) |
| **Interventions** |  |  |  |  |  |
| **RDN+WM vs** |  |  |  |  |  |
| SHL+WM | 0.98 (0.86, 1.10) | -0.31 (-1.31, 0.69) | 0.04 (-0.83, 0.90) | -- | -0.35 (-1.66, 0.97) |
| TRQ+WM | 1.01 (0.95, 1.05) | -0.49 (-1.16, 0.16) | -0.29 (-0.99, 0.39) | -0.41 (-1.89, 1.08) | -0.73 (-1.68, 0.21) |
| XBJ+WM | 0.89 (0.77, 1.00) | 0.02 (-1.11, 1.13) | -0.40 (-1.58, 0.76) | -0.75 (-2.25, 0.75) | 1.00 (-0.32, 2.32) |
| XYP+WM | **0.96 (0.92, 0.99)** | -0.45 (-1.03, 0.12) | -0.29 (-0.86, 0.28) | -0.49 (-1.23, 0.28) | -0.43 (-1.21, 0.35) |
| YHN+WM | 0.98 (0.90, 1.06) | -0.10 (-1.26, 1.04) | -- | 0.55 (-1.02, 2.12) | 0.13 (-1.66, 1.92) |
| WM | **1.14 (1.11, 1.18)** | **-1.58 (-2.03, -1.14)** | **-1.40 (-1.89, -0.93)** | **-1.25 (-1.82, -0.68)** | **-1.37 (-1.91, -0.83)** |
| **SHL+WM vs** |  |  |  |  |  |
| TRQ+WM | 1.02 (0.90, 1.17) | -0.19 (-1.21, 0.84) | -0.33 (-1.20, 0.55) | -- | -0.39 (-1.81, 1.04) |
| XBJ+WM | 0.91 (0.76, 1.08) | 0.32 (-1.04, 1.69) | -0.44 (-1.73, 0.85) | -- | 1.34 (-0.35, 3.05) |
| XYP+WM | 0.98 (0.87, 1.11) | -0.14 (-1.11, 0.83) | -0.33 (-1.10, 0.46) | -- | -0.08 (-1.40, 1.25) |
| YHN+WM | 0.99 (0.87, 1.16) | 0.20 (-1.18, 1.60) | -- | -- | 0.48 (-1.61, 2.56) |
| WM | **1.17 (1.04, 1.33)** | **-1.27 (-2.17, -0.37)** | **-1.44 (-2.16, -0.72)** | -- | -1.02 (-2.22, 0.18) |
| **TRQ+WM vs** |  |  |  |  |  |
| XBJ+WM | 0.89 (0.77, 1.01) | 0.51 (-0.63, 1.64) | -0.11 (-1.28, 1.07) | -0.34 (-2.29, 1.61) | **1.73 (0.31, 3.17)** |
| XYP+WM | 0.96 (0.91, 1.01) | 0.04 (-0.57, 0.66) | 0.01 (-0.57, 0.59) | -0.09 (-1.52, 1.39) | 0.30 (-0.65, 1.27) |
| YHN+WM | 0.98 (0.90, 1.07) | 0.39 (-0.78, 1.56) | -- | 0.96 (-1.05, 2.96) | 0.87 (-1.00, 2.74) |
| WM | **1.14 (1.10, 1.19)** | **-1.09 (-1.58, -0.60)** | **-1.11 (-1.60, -0.61)** | -0.84 (-2.21, 0.53) | -0.63 (-1.40, 0.14) |
| **XBJ+WM vs** |  |  |  |  |  |
| XYP+WM | 1.08 (0.96, 1.24) | -0.47 (-1.56, 0.62) | 0.12 (-0.99, 1.23) | 0.26 (-1.19, 1.74) | **-1.43 (-2.75, -0.10)** |
| YHN+WM | 1.11 (0.96, 1.29) | -0.12 (-1.59, 1.36) | -- | 1.30 (-0.72, 3.31) | -0.87 (-2.95, 1.22) |
| WM | **1.29 (1.14, 1.48)** | **-1.6 (-2.62, -0.57)** | -1.00 (-2.07, 0.07) | -0.50 (-1.88, 0.88) | **-2.36 (-3.57, -1.17)** |
| **XYP+WM vs** |  |  |  |  |  |
| YHN+WM | 1.03 (0.94, 1.11) | 0.35 (-0.77, 1.47) | -- | 1.04 (-0.52, 2.56) | 0.56 (-1.24, 2.36) |
| WM | **1.19 (1.16, 1.23)** | **-1.13 (-1.5, -0.76)** | **-1.12 (-1.42, -0.81)** | **-0.76 (-1.27, -0.28)** | **-0.94 (-1.50, -0.38)** |
| **YHN+WM vs** |  |  |  |  |  |
| WM | **1.16 (1.08, 1.26)** | **-1.48 (-2.54, -0.42)** | -- | **-1.80 (-3.26, -0.33)** | -1.50 (-3.20, 0.21) |

Note: underlined and bold results indicate statistically significant differences; red results were different from the overall results; RR, Risk Ratio; MD, Mean Differences; 95% CI, 95% Confidence Interval; WM, Western Medicine; RDN, Reduning injection; TRQ, Tanreqing injection; XBJ, Xuebijing injection; SHL, Shuanghuanglian injection; YHN, Yanhuning injection; XYP, Xiyanping injection.

# File S12: Subgroup of paediatric patients.

| **Interventions** | **Clinical effectiveness rate, RR (95% CI)** | **Antipyretic time, MD (95% CI)** | **Sore throat relief time, MD (95% CI)** | **Red and swollen tonsils relief time, MD (95% CI)** | **Tonsillar exudate relief time, MD (95% CI)** |
| --- | --- | --- | --- | --- | --- |
| **Ranking probabilities of SUCRA** | XBJ+WM ranked first (78.30%)/QKL+WM was not included in this outcome analysis | RDN+WM ranked first (68.08%) | SHL+WM ranked first (72.23%) | YHN+WM ranked first (91.09%) | XBJ+WM ranked first (94.86%) |
| **Interventions** |  |  |  |  |  |
| **CHN+WM vs** |  |  |  |  |  |
| RDN+WM | **0.02 (0.01, 0.72)** | -- | -- | -- | -- |
| SHL+WM | **0.02 (0.01, 0.69)** | -- | -- | -- | -- |
| TRQ+WM | **0.02 (0.01, 0.71)** | -- | -- | -- | -- |
| XBJ+WM | **0.02 (0.01, 0.67)** | -- | -- | -- | -- |
| XYP+WM | **0.02 (0.01, 0.69)** | -- | -- | -- | -- |
| YHN+WM | **0.02 (0.01, 0.70)** | -- | -- | -- | -- |
| YXC+WM | **0.02 (0.01, 0.72)** | -- | -- | -- | -- |
| WM | **0.02 (0.01, 0.79)** | -- | -- | -- | -- |
| **QKL+WM vs** |  |  |  |  |  |
| RDN+WM | -- | -0.09 (-1.69, 1.52) | -- | -- | 0.66 (-1.11, 2.41) |
| SHL+WM | -- | -0.29 (-2.03, 1.45) | -- | -- | 0.22 (-1.72, 2.15) |
| TRQ+WM | -- | -0.63 (-2.30, 1.04) | -- | -- | -0.23 (-2.12, 1.65) |
| XBJ+WM | -- | -0.07 (-1.95, 1.82) | -- | -- | 1.66 (-0.38, 3.72) |
| XYP+WM | -- | -0.50 (-2.09, 1.10) | -- | -- | 0.24 (-1.53, 2.01) |
| YHN+WM | -- | -0.19 (-2.09, 1.72) | -- | -- | 0.80 (-1.57, 3.17) |
| YXC+WM | -- | 0.00 (-2.18, 2.18) | -- | -- | 0.01 (-2.37, 2.37) |
| WM | -- | **-1.67 (-3.21, -0.13)** | -- | -- | -0.70 (-2.38, 0.98) |
| **RDN+WM vs** |  |  |  |  |  |
| SHL+WM | 1.04 (0.30, 3.06) | -0.19 (-1.12, 0.72) | 0.03 (-0.95, 0.99) | -- | -0.44 (-1.53, 0.66) |
| TRQ+WM | 0.99 (0.53, 1.84) | -0.54 (-1.33, 0.24) | -0.10 (-1.14, 0.95) | -0.41 (-1.70, 0.88) | -0.88 (-1.88, 0.11) |
| XBJ+WM | 0.63 (0.19, 1.72) | 0.02 (-1.16, 1.19) | -0.41 (-1.74, 0.93) | -0.75 (-2.05, 0.56) | 1.01 (-0.27, 2.30) |
| XYP+WM | 0.79 (0.51, 1.22) | -0.41 (-1.03, 0.20) | -0.29 (-0.95, 0.37) | -0.64 (-1.30, 0.04) | -0.41 (-1.17, 0.35) |
| YHN+WM | 1.34 (0.72, 2.45) | -0.10 (-1.30, 1.10) | -- | 0.55 (-0.84, 1.93) | 0.15 (-1.61, 1.89) |
| YXC+WM | 0.64 (0.19, 1.79) | 0.09 (-1.52, 1.69) | -- |  | -0.65 (-2.41, 1.10) |
| WM | **5.43 (3.86, 7.80)** | **-1.58 (-2.03, -1.13)** | **-1.41 (-1.96, -0.86)** | **-1.25 (-1.75, -0.75)** | **-1.35 (-1.88, -0.83)** |
| **SHL+WM vs** |  |  |  |  |  |
| TRQ+WM | 0.94 (0.31, 3.32) | -0.34 (-1.37, 0.69) | -0.13 (-1.31, 1.07) | -- | -0.45 (-1.73, 0.84) |
| XBJ+WM | 0.60 (0.13, 2.70) | 0.21 (-1.13, 1.57) | -0.44 (-1.88, 1.03) | -- | 1.45 (-0.07, 2.97) |
| XYP+WM | 0.75 (0.27, 2.53) | -0.21 (-1.12, 0.70) | -0.32 (-1.19, 0.57) | -- | 0.02 (-1.08, 1.13) |
| YHN+WM | 1.27 (0.41, 4.66) | 0.10 (-1.27, 1.48) | -- | -- | 0.58 (-1.35, 2.51) |
| YXC+WM | 0.61 (0.13, 2.81) | 0.29 (-1.45, 2.02) | -- | -- | -0.22 (-2.14, 1.71) |
| WM | **5.19 (1.92, 17.42)** | **-1.38 (-2.18, -0.57)** | **-1.44 (-2.23, -0.63)** | -- | -0.92 (-1.88, 0.04) |
| **TRQ+WM vs** |  |  |  |  |  |
| XBJ+WM | 0.63 (0.19, 1.83) | 0.56 (-0.70, 1.82) | -0.31 (-1.81, 1.20) | -0.34 (-2.03, 1.35) | **1.89 (0.45, 3.35)** |
| XYP+WM | 0.79 (0.46, 1.41) | 0.13 (-0.64, 0.90) | -0.19 (-1.15, 0.76) | -0.23 (-1.50, 1.05) | 0.47 (-0.54, 1.48) |
| YHN+WM | 1.35 (0.66, 2.79) | 0.44 (-0.85, 1.74) | -- | 0.96 (-0.80, 2.72) | 1.03 (-0.84, 2.90) |
| YXC+WM | 0.64 (0.18, 1.99) | 0.63 (-1.03, 2.29) | -- | -- | 0.23 (-1.64, 2.11) |
| WM | **5.47 (3.35, 9.30)** | **-1.04 (-1.68, -0.40)** | **-1.31 (-2.20, -0.43)** | -0.84 (-2.03, 0.35) | -0.47 (-1.32, 0.38) |
| **XBJ+WM vs** |  |  |  |  |  |
| XYP+WM | 1.25 (0.47, 3.96) | -0.43 (-1.59, 0.73) | 0.12 (-1.16, 1.39) | 0.11 (-1.17, 1.41) | **-1.43 (-2.72, -0.13)** |
| YHN+WM | 2.13 (0.74, 7.27) | -0.12 (-1.67, 1.44) | -- | 1.30 (-0.47, 3.07) | -0.86 (-2.91, 1.17) |
| YXC+WM | 1.01 (0.23, 4.50) | 0.07 (-1.81, 1.95) | -- | -- | -1.66 (-3.71, 0.38) |
| WM | **8.64 (3.42, 26.89)** | **-1.6 (-2.68, -0.51)** | -1.00 (-2.22, 0.22) | -0.50 (-1.71, 0.71) | **-2.36 (-3.54, -1.19)** |
| **XYP+WM vs** |  |  |  |  |  |
| YHN+WM | 1.70 (0.95, 2.97) | 0.31 (-0.88, 1.51) | -- | 1.18 (-0.19, 2.55) | 0.56 (-1.20, 2.32) |
| YXC+WM | 0.81 (0.24, 2.27) | 0.50 (-1.10, 2.09) | -- | -- | -0.24 (-2.00, 1.52) |
| WM | **6.92 (5.31, 9.02)** | **-1.17 (-1.59, -0.75)** | **-1.12 (-1.49, -0.75)** | **-0.61 (-1.07, -0.17)** | **-0.94 (-1.49, -0.39)** |
| **YHN+WM vs** |  |  |  |  |  |
| YXC+WM | 0.48 (0.13, 1.42) | 0.19 (-1.71, 2.09) | -- | -- | -0.80 (-3.16, 1.57) |
| WM | **4.07 (2.51, 6.77)** | **-1.48 (-2.60, -0.36)** | -- | **-1.80 (-3.09, -0.51)** | -1.50 (-3.17, 0.17) |
| **YXC+WM vs** |  |  |  |  |  |
| WM | **8.53 (3.20, 27.54)** | **-1.67 (-3.20, -0.13)** | -- | -- | -0.70 (-2.37, 0.97) |

Note: underlined and bold results indicate statistically significant differences; red results were different from the overall results; RR, Risk Ratio; MD, Mean Differences; 95% CI, 95% Confidence Interval; WM, Western Medicine; RDN, Reduning injection; TRQ, Tanreqing injection; XBJ, Xuebijing injection; SHL, Shuanghuanglian injection; YHN, Yanhuning injection; CHN, Chuanhuning injection; XYP, Xiyanping injection; YXC, Yuxingcao injection.

# File S13: Subgroup of patients with suppurative tonsillitis.

|  | **Clinical effectiveness rate, RR (95% CI)** | **Antipyretic time, MD (95% CI)** | **Sore throat relief time, MD (95% CI)** | **Red and swollen tonsils relief time, MD (95% CI)** | **Tonsillar exudate relief time, MD (95% CI)** |
| --- | --- | --- | --- | --- | --- |
| **Ranking probabilities of SUCRA** | QKL+WM ranked first (81.88%) | XBJ+WM ranked first (94.87%) | SHL+WM ranked first (74.54%) | YHN+WM ranked first (91.47%) | XBJ+WM ranked first (94.80%) |
| **Interventions** |  |  |  |  |  |
| **CHN+WM vs** |  |  |  |  |  |
| QKL+WM | **0.02 (0.01, 0.60)** | -- | -- | -- | -- |
| RDN+WM | **0.02 (0.01, 0.79)** | -- | -- | -- | -- |
| SHL+WM | **0.02 (0.01, 0.68)** | -- | -- | -- | -- |
| TRQ+WM | **0.03 (0.01, 0.67)** | -- | -- | -- | -- |
| XBJ+WM | **0.02 (0.01, 0.63)** | -- | -- | -- | -- |
| XYP+WM | **0.02 (0.01, 0.65)** | -- | -- | -- | -- |
| YHN+WM | **0.02 (0.01, 0.73)** | -- | -- | -- | -- |
| YXC+WM | **0.02 (0.01, 0.71)** | -- | -- | -- | -- |
| WM | **0.02 (0.01, 0.85)** | -- | -- | -- | -- |
| **QKL+WM vs** |  |  |  |  |  |
| RDN+WM | 1.10 (0.91, 1.41) | 0.65 (-1.11, 2.41) | -- | -- | 0.73 (-1.00, 2.47) |
| SHL+WM | 1.12 (0.90, 1.41) | 0.22 (-1.72, 2.15) | -- | -- | 0.22 (-1.70, 2.14) |
| TRQ+WM | 1.12 (0.97, 1.36) | -0.23 (-2.11, 1.66) | -- | -- | -0.06 (-1.88, 1.76) |
| XBJ+WM | 1.01 (0.82, 1.28) | 1.66 (-0.38, 3.72) | -- | -- | 1.66 (-0.36, 3.70) |
| XYP+WM | 1.09 (0.94, 1.33) | 0.24 (-1.53, 2.01) | -- | -- | 0.24 (-1.5, 2.00) |
| YHN+WM | 1.11 (0.91, 1.35) | 0.8 (-1.57, 3.17) | -- | -- | 0.80 (-1.54, 3.15) |
| YXC+WM | 1.13 (0.89, 1.39) | 0.01 (-2.37, 2.37) | -- | -- | 0.01 (-2.34, 2.35) |
| WM | **1.31 (1.11, 1.57)** | -0.7 (-2.38, 0.98) | -- | -- | -0.70 (-2.36, 0.97) |
| **RDN+WM vs** |  |  |  |  |  |
| SHL+WM | 0.97 (0.85, 1.11) | -0.44 (-1.53, 0.66) | 0.04 (-0.88, 0.93) | -- | -0.51 (-1.58, 0.56) |
| TRQ+WM | 0.99 (0.93, 1.05) | -0.88 (-1.89, 0.12) | -0.15 (-0.88, 0.57) | -0.44 (-1.66, 0.77) | -0.79 (-1.69, 0.10) |
| XBJ+WM | 0.90 (0.75, 1.01) | 1.01 (-0.28, 2.31) | -0.40 (-1.64, 0.83) | -0.78 (-2.01, 0.44) | 0.94 (-0.32, 2.20) |
| XYP+WM | **0.95 (0.94, 0.99)** | -0.41 (-1.18, 0.35) | -0.29 (-0.90, 0.32) | **-0.67 (-1.28, -0.06)** | -0.49 (-1.23, 0.25) |
| YHN+WM | 0.97 (0.91, 1.06) | 0.14 (-1.61, 1.90) | -- | 0.52 (-0.80, 1.83) | 0.07 (-1.65, 1.79) |
| YXC+WM | 0.99 (0.90, 1.07) | -0.65 (-2.41, 1.10) | -- |  | -0.73 (-2.46, 1.00) |
| WM | **1.14 (1.12, 1.16)** | **-1.35 (-1.88, -0.83)** | **-1.40 (-1.91, -0.90)** | **-1.28 (-1.72, -0.85)** | **-1.43 (-1.93, -0.93)** |
| **SHL+WM vs** |  |  |  |  |  |
| TRQ+WM | 1.01 (0.89, 1.15) | -0.44 (-1.73, 0.84) | -0.19 (-1.09, 0.73) | -- | -0.28 (-1.49, 0.92) |
| XBJ+WM | 0.92 (0.75, 1.06) | 1.45 (-0.07, 2.98) | -0.44 (-1.78, 0.92) | -- | 1.45 (-0.05, 2.95) |
| XYP+WM | 0.97 (0.85, 1.12) | 0.02 (-1.09, 1.13) | -0.32 (-1.14, 0.51) | -- | 0.02 (-1.07, 1.12) |
| YHN+WM | 0.99 (0.85, 1.14) | 0.58 (-1.34, 2.51) | -- | -- | 0.58 (-1.32, 2.49) |
| YXC+WM | 1.01 (0.86, 1.17) | -0.22 (-2.15, 1.71) | -- | -- | -0.22 (-2.13, 1.69) |
| WM | **1.16 (1.03, 1.31)** | -0.92 (-1.88, 0.05) | **-1.44 (-2.18, -0.69)** | -- | -0.92 (-1.87, 0.03) |
| **TRQ+WM vs** |  |  |  |  |  |
| XBJ+WM | 0.90 (0.75, 1.01) | **1.89 (0.45, 3.35)** | -0.25 (-1.48, 0.99) | -0.34 (-1.94, 1.27) | **1.73 (0.35, 3.12)** |
| XYP+WM | 0.94 (0.93, 1.01) | 0.47 (-0.54, 1.48) | -0.14 (-0.75, 0.49) | -0.23 (-1.43, 0.99) | 0.30 (-0.62, 1.23) |
| YHN+WM | 0.95 (0.91, 1.05) | 1.02 (-0.85, 2.90) | -- | 0.96 (-0.71, 2.64) | 0.86 (-0.95, 2.68) |
| YXC+WM | 0.90 (0.89, 1.10) | 0.23 (-1.65, 2.11) | -- | -- | 0.06 (-1.75, 1.88) |
| WM | **1.13 (1.12, 1.19)** | -0.47 (-1.32, 0.38) | **-1.25 (-1.77, -0.73)** | -0.84 (-1.97, 0.29) | -0.64 (-1.38, 0.11) |
| **XBJ+WM vs** |  |  |  |  |  |
| XYP+WM | 1.07 (0.94, 1.25) | **-1.42 (-2.73, -0.13)** | 0.11 (-1.06, 1.29) | 0.11 (-1.11, 1.34) | **-1.43 (-2.71, -0.15)** |
| YHN+WM | 1.11 (0.93, 1.30) | -0.87 (-2.91, 1.17) | -- | 1.30 (-0.38, 2.98) | -0.87 (-2.89, 1.15) |
| YXC+WM | 1.09 (0.90, 1.31) | -1.67 (-3.72, 0.37) | -- | -- | -1.66 (-3.70, 0.35) |
| WM | **1.24 (1.13, 1.46)** | **-2.36 (-3.54, -1.19)** | -1.00 (-2.12, 0.12) | -0.50 (-1.64, 0.64) | **-2.36 (-3.53, -1.21)** |
| **XYP+WM vs** |  |  |  |  |  |
| YHN+WM | 1.01 (0.94, 1.10) | 0.56 (-1.20, 2.31) | -- | 1.19 (-0.13, 2.49) | 0.56 (-1.18, 2.29) |
| YXC+WM | 1.03 (0.92, 1.11) | -0.24 (-2.00, 1.52) | -- | -- | -0.24 (-1.99, 1.50) |
| WM | **1.22 (1.12, 1.23)** | **-0.94 (-1.49, -0.39)** | **-1.12 (-1.46, -0.78)** | **-0.61 (-1.05, -0.19)** | **-0.94 (-1.48, -0.39)** |
| **YHN+WM vs** |  |  |  |  |  |
| YXC+WM | 1.02 (0.90, 1.15) | -0.80 (-3.16, 1.57) | -- | -- | -0.80 (-3.14, 1.53) |
| WM | **1.18** **(1.13, 1.25)** | -1.50 (-3.17, 0.17) | -- | **-1.80 (-3.04, -0.56)** | -1.50 (-3.15, 0.15) |
| **YXC+WM vs** |  |  |  |  |  |
| WM | **1.15 (1.05, 1.30)** | -0.70 (-2.37, 0.98) | -- | -- | -0.70 (-2.35, 0.96) |

Note: underlined and bold results indicate statistically significant differences; red results were different from the overall results; RR, Risk Ratio; MD, Mean Differences; 95% CI, 95% Confidence Interval; WM, Western Medicine; RDN, Reduning injection; TRQ, Tanreqing injection; QKL, Qingkailing injection; XBJ, Xuebijing injection; SHL, Shuanghuanglian injection; YHN, Yanhuning injection; CHN, Chuanhuning injection; XYP, Xiyanping injection; YXC, Yuxingcao injection.

# File S14: Subgroup for patients who received penicillins as the treatment regimen of WM.

|  | **Clinical effectiveness rate, RR (95% CI)** | **Antipyretic time, MD (95% CI)** | **Sore throat relief time, MD (95% CI)** | **Red and swollen tonsils relief time, MD (95% CI)** | **Tonsillar exudate relief time, MD (95% CI)** |
| --- | --- | --- | --- | --- | --- |
| **Ranking probabilities of SUCRA** | QKL+WM ranked first (84.87%) | RDN+WM ranked first (63.93%) | SHL+WM ranked first (76.34%) | XYP+WM ranked first (81.19%) | YHN+WM ranked first (89.78%)/XBJ+WM was not included in this outcome analysis |
| **Interventions** |  |  |  |  |  |
| **QKL+WM vs** |  |  |  |  |  |
| RDN+WM | 1.12 (0.9, 1.46) | -0.11 (-2.31, 2.11) | -- | -- | 0.36 (-0.77, 1.54) |
| SHL+WM | 1.11 (0.86, 1.47) | -0.29 (-2.63, 2.04) | -- | -- | 0.24 (-1.01, 1.45) |
| TRQ+WM | 1.1 (0.87, 1.44) | -0.39 (-2.68, 1.9) | -- | -- | -0.81 (-2.17, 0.47) |
| XYP+WM | 1.14 (0.9, 1.49) | -0.53 (-3.5, 2.44) | -- | -- | -- |
| YHN+WM | 1.12 (0.88, 1.46) | -0.18 (-2.74, 2.38) | -- | -- | 0.8 (-0.7, 2.3) |
| YXC+WM | 1.14 (0.89, 1.5) | 0 (-2.94, 2.95) | -- | -- | 0.1 (-1.51, 1.51) |
| WM | **1.29 (1.03, 1.68)** | -1.67 (-3.75, 0.41) | -- | -- | -0.7 (-1.77, 0.37) |
| **RDN+WM vs** |  |  |  |  |  |
| SHL+WM | 0.98 (0.87, 1.1) | -0.18 (-1.49, 1.1) | 0.16 (-0.44, 0.66) | -- | -0.12 (-0.91, 0.58) |
| TRQ+WM | 0.98 (0.92, 1.04) | -0.28 (-1.48, 0.91) | 0.13 (-0.39, 0.58) | -- | **-1.17 (-2.12, -0.34)** |
| XYP+WM | 1.01 (0.96, 1.07) | -0.42 (-2.66, 1.81) | -0.13 (-0.91, 0.6) | 0.85 (-1.75, 3.37) | -- |
| YHN+WM | 0.99 (0.92, 1.07) | -0.07 (-1.75, 1.58) | -- | 0.41 (-2.12, 2.88) | 0.44 (-0.72, 1.55) |
| YXC+WM | 1.01 (0.91, 1.11) | 0.12 (-2.1, 2.31) | -- | -- | -0.36 (-1.54, 0.76) |
| WM | **1.15 (1.11, 1.19)** | **-1.55 (-2.31, -0.83)** | **-1.36 (-1.65, -1.13)** | **-1.39 (-2.65, -0.18)** | **-1.06 (-1.52, -0.65)** |
| **SHL+WM vs** |  |  |  |  |  |
| TRQ+WM | 0.99 (0.88, 1.14) | -0.1 (-1.52, 1.32) | -0.04 (-0.63, 0.63) | -- | **-1.05 (-2.05, -0.09)** |
| XYP+WM | 1.03 (0.92, 1.17) | -0.24 (-2.6, 2.13) | -0.3 (-1.12, 0.59) | -- | -- |
| YHN+WM | 1.01 (0.89, 1.16) | 0.11 (-1.72, 1.94) | -- | -- | 0.56 (-0.64, 1.79) |
| YXC+WM | 1.03 (0.89, 1.19) | 0.29 (-2.04, 2.63) | -- | -- | -0.24 (-1.44, 1) |
| WM | **1.17 (1.05, 1.32)** | **-1.38 (-2.44, -0.31)** | **-1.53 (-1.98, -1.02)** |  | **-0.94 (-1.53, -0.32)** |
| **TRQ+WM vs** |  |  |  |  |  |
| XYP+WM | 1.04 (0.96, 1.11) | -0.14 (-2.45, 2.17) | -0.26 (-1.08, 0.56) | -- | -- |
| YHN+WM | 1.02 (0.93, 1.11) | 0.21 (-1.55, 1.97) | -- | -- | 1.61 (0.34, 2.95) |
| YXC+WM | 1.04 (0.92, 1.15) | 0.39 (-1.89, 2.68) | -- | -- | 0.81 (-0.47, 2.16) |
| WM | **1.17 (1.11, 1.24)** | **-1.28 (-2.22, -0.34)** | **-1.49 (-1.9, -1.07)** | -- | 0.11 (-0.63, 0.92) |
| **XYP+WM vs** |  |  |  |  |  |
| YHN+WM | 0.98 (0.9, 1.07) | 0.35 (-2.24, 2.92) | -- | -0.44 (-3.55, 2.68) | -- |
| YXC+WM | 1 (0.89, 1.11) | 0.53 (-2.43, 3.49) | -- | -- | -- |
| WM | **1.13 (1.08, 1.19)** | -1.14 (-3.25, 0.97) | **-1.23 (-1.94, -0.52)** | -2.24 (-4.48, 0.02) | -- |
| **YHN+WM vs** |  |  |  |  |  |
| YXC+WM | 1.02 (0.9, 1.14) | 0.19 (-2.37, 2.74) | -- | -- | -0.8 (-2.3, 0.69) |
| WM | **1.15 (1.08, 1.24)** | -1.48 (-2.98, 0.01) | -- | -1.8 (-3.97, 0.37) | **-1.5 (-2.55, -0.44)** |
| **YXC+WM vs** |  |  |  |  |  |
| WM | **1.13 (1.04, 1.26)** | -1.67 (-3.75, 0.41) | -- | -- | -0.7 (-1.76, 0.36) |

Note: underlined and bold results indicate statistically significant differences; red results were different from the overall results; RR, Risk Ratio; MD, Mean Differences; 95% CI, 95% Confidence Interval; WM, Western Medicine; RDN, Reduning injection; TRQ, Tanreqing injection; QKL, Qingkailing injection; YHN, Yanhuning injection; SHL, Shuanghuanglian injection; XYP, Xiyanping injection; YXC, Yuxingcao injection.

# File S15: Subgroup for patients who received cephalosporins as the treatment regimen of WM.

|  | **Clinical effectiveness rate, RR (95% CI)** | **Antipyretic time, MD (95% CI)** | **Sore throat relief time, MD (95% CI)** | **Red and swollen tonsils relief time, MD (95% CI)** | **Tonsillar exudate relief time, MD (95% CI)** |
| --- | --- | --- | --- | --- | --- |
| **Ranking probabilities of SUCRA** | XBJ+WM ranked first (99.35%) | XBJ+WM ranked first (95.41%) | TRQ+WM ranked first (77.97%)/ SHL+WM was not included in this outcome analysis | RDN+WM ranked first (85.13%)/ YHN+WM was not included in this outcome analysis | XBJ+WM ranked first (96.65%) |
| **Interventions** |  |  |  |  |  |
| **CHN+WM vs** |  |  |  |  |  |
| QKL+WM | **0.04 (0.01, 0.65)** | -- | -- | -- | -- |
| RDN+WM | **0.05 (0.01, 0.79)** | -- | -- | -- | -- |
| TRQ+WM | **0.05 (0.01, 0.78)** | -- | -- | -- | -- |
| XBJ+WM | **0.02 (0.01, 0.26)** | -- | -- | -- | -- |
| XYP+WM | **0.05 (0.01, 0.74)** | -- | -- | -- | -- |
| YHN+WM | **0.05 (0.01, 0.7)** | -- | -- | -- | -- |
| YXC+WM | **0.04 (0.01, 0.68)** | -- | -- | -- | -- |
| WM | **0.06 (0.01, 0.88)** | -- | -- | -- | -- |
| **QKL+WM vs** |  |  |  |  |  |
| RDN+WM | 1.23 (0.97, 1.64) | -- | -- | -- | -- |
| TRQ+WM | 1.21 (0.96, 1.62) | -- | -- | -- | -- |
| XBJ+WM | **0.08 (0.01, 0.99)** | -- | -- | -- | -- |
| XYP+WM | 1.15 (0.91, 1.55) | -- | -- | -- | -- |
| YHN+WM | 1.09 (0.82, 1.51) | -- | -- | -- | -- |
| YXC+WM | 1.04 (0.72, 1.49) | -- | -- | -- | -- |
| WM | **1.37 (1.09, 1.82)** | -- | -- | -- | -- |
| **RDN+WM vs** |  |  |  |  |  |
| TRQ+WM | 0.99 (0.91, 1.07) | **-0.8 (-1.47, -0.09)** | -- | -0.36 (-2.11, 1.41) | -0.53 (-2.31, 1.24) |
| XBJ+WM | **0.07 (0.01, 0.78)** | 0.51 (-0.57, 1.62) | -- | -- | 1.89 (-0.46, 4.22) |
| XYP+WM | 0.94 (0.85, 1.04) | -0.7 (-1.41, 0.05) | -- | -- | -0.11 (-1.84, 1.58) |
| YHN+WM | 0.89 (0.74, 1.06) | -- | -- | -- | -- |
| YXC+WM | 0.85 (0.63, 1.07) | -- | -- | -- | -- |
| WM | **1.11 (1.06, 1.18)** | **-1.69 (-2.17, -1.18)** |  | **-1.2 (-1.98, -0.39)** | **-1.81 (-2.76, -0.86)** |
| **TRQ+WM vs** |  |  |  |  |  |
| XBJ+WM | **0.07 (0.01, 0.79)** | **1.31 (0.22, 2.39)** | -- | -- | 2.42 (-0.19, 5.03) |
| XYP+WM | 0.95 (0.86, 1.05) | 0.1 (-0.61, 0.82) | -0.03 (-0.52, 0.51) | -- | 0.42 (-1.66, 2.47) |
| YHN+WM | 0.9 (0.75, 1.07) | -- | -- | -- | -- |
| YXC+WM | 0.86 (0.64, 1.08) | -- | -- | -- | -- |
| WM | **1.13 (1.07, 1.2)** | **-0.89 (-1.37, -0.41)** | **-1.05 (-1.35, -0.71)** | -0.84 (-2.41, 0.73) | -1.28 (-2.78, 0.21) |
| **XBJ+WM vs** |  |  |  |  |  |
| XYP+WM | **13.67 (1.2, 5548.79)** | **-1.21 (-2.31, -0.1)** | -- | -- | -2 (-4.57, 0.55) |
| YHN+WM | **12.96 (1.11, 5250.26)** | -- | -- | -- | -- |
| YXC+WM | **12.28 (1.04, 5065.86)** | -- | -- | -- | -- |
| WM | **16.24 (1.42, 6532.49)** | **-2.2 (-3.17, -1.23)** | -- | -- | **-3.7 (-5.84, -1.56)** |
| **XYP+WM vs** |  |  |  |  |  |
| YHN+WM | 0.95 (0.78, 1.13) | -- | -- | -- | -- |
| YXC+WM | 0.9 (0.66, 1.15) | -- | -- | -- | -- |
| WM | **1.18 (1.09, 1.29)** | **-0.99 (-1.53, -0.47)** | **-1.02 (-1.43, -0.61)** | -- | **-1.7 (-3.11, -0.26)** |
| **YHN+WM vs** |  |  |  |  |  |
| YXC+WM | 0.95 (0.68, 1.27) | -- | -- | -- | -- |
| WM | **1.25 (1.07, 1.49)** | -- | -- | -- | -- |
| **YXC+WM vs** |  |  |  |  |  |
| WM | **1.32 (1.05, 1.77)** | -- | -- | -- | -- |

Note: underlined and bold results indicate statistically significant differences; red results were different from the overall results; RR, Risk Ratio; MD, Mean Differences; 95% CI, 95% Confidence Interval; WM, Western Medicine; RDN, Reduning injection; TRQ, Tanreqing injection; QKL, Qingkailing injection; XBJ, Xuebijing injection; YHN, Yanhuning injection; CHN, Chuanhuning injection; XYP, Xiyanping injection; YXC, Yuxingcao injection.
